# Role of the kisspeptin-KISS1R axis in the pathogenesis of chronic kidney disease and uremic cardiomyopathy

**DOI:** 10.1007/s11357-023-01017-8

**Published:** 2023-11-21

**Authors:** Hoa Dinh, Zsuzsanna Z. A. Kovács, Merse Kis, Klaudia Kupecz, Anita Sejben, Gergő Szűcs, Fanni Márványkövi, Andrea Siska, Marah Freiwan, Szonja Polett Pósa, Zsolt Galla, Katalin Eszter Ibos, Éva Bodnár, Gülsüm Yilmaz Lauber, Ana Isabel Antunes Goncalves, Eylem Acar, András Kriston, Ferenc Kovács, Péter Horváth, Zsolt Bozsó, Gábor Tóth, Imre Földesi, Péter Monostori, Gábor Cserni, Bruno K. Podesser, Andrea Lehoczki, Peter Pokreisz, Attila Kiss, László Dux, Krisztina Csabafi, Márta Sárközy

**Affiliations:** 1 Department of Biochemistry and Interdisciplinary Centre of Excellence, Albert Szent-Györgyi Medical School, University of Szeged, 6720 Szeged, Hungary; 2 Department of Biochemistry, Bach Mai Hospital, Hanoi, 100000 Vietnam; 3 Department of Pathophysiology, Albert Szent-Györgyi Medical School, University of Szeged, Szeged, 6720 Hungary; 4 Department of Pathology, Albert Szent-Györgyi Medical School, University of Szeged, Szeged, 6720 Hungary; 5 Department of Laboratory Medicine, Albert Szent-Györgyi Medical School, University of Szeged, 6720 Szeged, Hungary; 6 Metabolic and Newborn Screening Laboratory, Department of Pediatrics, Albert Szent-Györgyi Medical School, University of Szeged, 6720 Szeged, Hungary; 7 Ludwig Boltzmann Institute for Cardiovascular Research at Center for Biomedical Research and Translational Surgery, Medical University of Vienna, 1090 Vienna, Austria; 8 Synthetic and Systems Biology Unit, Biological Research Centre, Eötvös Loránd Research Network, 6726 Szeged, Hungary; 9 Single-Cell Technologies Ltd, Szeged, 6726 Hungary; 10 Institute for Molecular Medicine Finland (FIMM), University of Helsinki, 00014 Helsinki, Finland; 11 Department of Medical Chemistry, Albert Szent-Györgyi Medical School, University of Szeged, 6720 Szeged, Hungary; 12 Departments of Hematology and Stem Cell Transplantation, South Pest Central Hospital, National Institute of Hematology and Infectious Diseases, Saint Ladislaus Campus, Budapest, Hungary

**Keywords:** Chronic renal failure, Kisspeptin-13, Hypertension, Cardiac hypertrophy, Inflammaging, Fibrosis, Apoptosis

## Abstract

The prevalence of chronic kidney disease (CKD) is increasing globally, especially in elderly patients. Uremic cardiomyopathy is a common cardiovascular complication of CKD, characterized by left ventricular hypertrophy (LVH), diastolic dysfunction, and fibrosis. Kisspeptins and their receptor, KISS1R, exert a pivotal influence on kidney pathophysiology and modulate age-related pathologies across various organ systems. KISS1R agonists, including kisspeptin-13 (KP-13), hold promise as novel therapeutic agents within age-related biological processes and kidney-related disorders. Our investigation aimed to elucidate the impact of KP-13 on the trajectory of CKD and uremic cardiomyopathy. Male Wistar rats (300–350 g) were randomized into four groups: (I) sham-operated, (II) 5/6 nephrectomy-induced CKD, (III) CKD subjected to a low dose of KP-13 (intraperitoneal 13 µg/day), and (IV) CKD treated with a higher KP-13 dose (intraperitoneal 26 µg/day). Treatments were administered daily from week 3 for 10 days. After 13 weeks, KP-13 increased systemic blood pressure, accentuating diastolic dysfunction’s echocardiographic indicators and intensifying CKD-associated markers such as serum urea levels, glomerular hypertrophy, and tubular dilation. Notably, KP-13 did not exacerbate circulatory uremic toxin levels, renal inflammation, or fibrosis markers. In contrast, the higher KP-13 dose correlated with reduced posterior and anterior wall thickness, coupled with diminished cardiomyocyte cross-sectional areas and concurrent elevation of inflammatory (*Il6, Tnf*), fibrosis (*Col1*), and apoptosis markers (*Bax/Bcl2*) relative to the CKD group. In summary, KP-13’s influence on CKD and uremic cardiomyopathy encompassed heightened blood pressure and potentially activated inflammatory and apoptotic pathways in the left ventricle.

**Supplementary Information:**

The online version contains supplementary material available at 10.1007/s11357-023-01017-8.

## Introduction

Chronic kidney disease (CKD) is a significant health concern affecting 7–12% of the general population that exhibits a notable increase in prevalence with aging [1, 2]. As individuals age, the risk of developing CKD substantially rises. Indeed, around half of the population over 70 years of age have CKD based on the estimated glomerular filtration rate (GFR < 60 mL/min/1.73 m^2^) [3]. This age-associated rise in CKD prevalence can be attributed to a combination of factors, including an increasing prevalence of risk factors and comorbidities (such as hypertension, obesity, diabetes mellitus, and atherosclerosis [3]) and the cumulative effects of molecular and cellular mechanisms of aging that contribute to the pathogenesis of the disease. One such mechanism is inflammaging, a state of chronic, low-grade inflammation that occurs with aging [4–6]. Inflammaging is characterized by elevated levels of pro-inflammatory cytokines and immune system dysregulation. In the context of CKD, inflammaging promotes renal tissue damage and fibrosis, impairing the kidney’s ability to function properly and contributing to the progressive decline of renal function over time [4, 5, 7].

Older patients with CKD and end-stage renal disease also have a 5–tenfold higher risk for cardiovascular diseases, including heart failure [8, 9]. Uremic cardiomyopathy is characterized by CKD-associated chronic and often irreversible structural and functional changes of the heart [10, 11] and is often accompanied by left ventricular hypertrophy (LVH), diastolic dysfunction, and cardiac fibrosis in CKD patients [8, 12, 13]. A synergistic interplay of mechanisms related to cardiac aging and CKD-associated factors collaboratively fosters the progression of uremic cardiomyopathy. This intricate convergence involves a spectrum of influences, encompassing non-CKD specific factors such as hypertension, hemodynamic overload, overactivation of the renin–angiotensin–aldosterone system (RAAS), sympathetic nervous system hyperactivity, endothelial dysfunction, and increased oxidative stress. Inflammation plays a pivotal role in the pathogenesis of uremic cardiomyopathy. Moreover, CKD-specific factors, including the presence of circulating uremic toxins and renal anemia, further compound the complex landscape that contributes to the development of this condition [8, 13]. Despite the widespread accessibility of conventional medications aimed at managing age-related comorbidities and risk factors, such as hypertension, obesity, and diabetes mellitus, the elevated cardiovascular morbidity and mortality observed among CKD patients persist as an unresolved challenge. As a result, there is a pressing need to identify novel therapeutic targets and agents capable of mitigating the severity of uremic cardiomyopathy. The pursuit of such innovative solutions holds significant promise in addressing this pressing medical concern.

Kisspeptins (KPs) and their receptor, KISS1R, have emerged as potential players in the pathogenesis of chronic kidney disease (CKD) as well as age-related pathologies of other organ systems [14–20]. Kisspeptins are proteins encoded by the *Kiss1* gene [21] that was initially discovered as a human metastasis suppressor gene in melanoma and several breast cancer cell lines [22]. The 145 amino acid-encoding product of the *Kiss1* gene is cleaved into shorter KPs of 54, 14, 13, or 10 amino acids in length [21]. Plasma levels of these C-terminal cleavage fragments, including KP-54 (formerly known as metastin), KP-14, KP-13, and KP-10, show age dependence [17, 23, 24]. All have biological activity and are the endogenous ligands for the G-protein-coupled receptor GPR54 (i.e., KISS1R) [17, 23, 24]. The kisspeptin-KISS1R system, initially recognized for its role in reproductive physiology, is now known to exert various functions in non-reproductive tissues, including the kidneys. Recent research has highlighted the presence of KISS1R in renal tissues and its involvement in regulating renal hemodynamics, electrolyte balance, and blood pressure [14, 25–27]. Dysregulation of the kisspeptin-KISS1R axis has been linked to impaired renal function and progression of CKD [14]. Importantly, in animal models of CKD (5/6 nephrectomized rats), KISS1R is significantly downregulated in the kidney [14].

Furthermore, studies suggest that kisspeptins might influence inflammatory responses in different organs, thereby implicating their potential role in age-related inflammation (inflammaging) seen in CKD [19, 20]. Moreover, recent research also demonstrate the kisspeptin-KISS1R axis also plays a role in the regulation of fibrotic processes and may modulate the genesis of cardiovascular pathologies. Indeed, we have recently published that the KISS1R antagonist peptide-234 hastened the development of uremic cardiomyopathy, probably, via activating the fibrotic transforming growth factor-beta (TGF-β)-mediated pathways [28]. Moreover, activating the KISS1R signaling pathway protects against liver steatosis, inflammation, and fibrosis in non-alcoholic steatohepatitis [29]. In bleomycin‑induced pulmonary hypertension, KP-13 was reported to reduce fibrosis by repressing tumor necrosis factor-α (*Tnf*), transforming growth factor-β (*Tgfb*), and collagen type Iα1 (*Col1a1*) [30]. In contrast, KP-10, KP-13, and KP-54 were shown to exert vasoconstrictive effects via the KISS1R in human coronary arteries and aorta [31]. Investigating the specific mechanisms by which kisspeptins and KISS1R contribute to the pathogenesis of CKD and uremic cardiomyopathy holds promise for identifying novel therapeutic targets and developing strategies to mitigate the progression of these debilitating conditions. However, further research is warranted to fully elucidate the intricate interplay of the kisspeptin-KISS1R system in CKD and its potential implications for future therapeutic interventions.

The current study was designed to investigate the hypothesis that pharmacological activation of the kisspeptin-KISS1R system could potentially confer anti-aging, anti-inflammatory, and anti-fibrotic effects [30], thereby offering mitigation against the progression of both uremic cardiomyopathy and renal failure in the context of CKD. To assess this hypothesis, we utilized a thoroughly characterized preclinical model of CKD and subsequently evaluated the impact of treatment with the KISS1R agonist KP-13 on both cardiac and renal outcomes. Serum and urine parameters were measured to estimate kidney function, echocardiography was performed to monitor cardiac function and morphology, renal angiotensin convertase enzyme (ACE) activity and blood pressure measurements were performed to evaluate hypertension, histology was performed to assess renal and left ventricular hypertrophy and fibrosis, and RT-qPCR and Western blot were performed to detect the gene expression changes of selected hypertrophy, inflammatory, and fibrotic markers both in the kidney and left ventricular samples.

## Materials and methods

### Ethics approval

This investigation conformed to the EU Directive 2010/63/EU and was approved by the regional Animal Research Ethics Committee of Csongrád County (XV.2598/2020, date of approval: 18 September 2020) and the University of Szeged in Hungary. All institutional and national guidelines for the care and use of laboratory animals were followed.

### Experimental animals

In the present study, the used 32 male Wistar rats (*Rattus norvegicus*, 300–350 g, 8 weeks old) were housed in pairs in individually ventilated cages (Tecniplast Sealsafe IVC system, Buguggiate, Italy) in a temperature-controlled room (22 ± 2 °C; relative humidity 55 ± 10%) with a 12 h:12 h light/dark cycle. Standard rat chow and tap water were supplied ad libitum. After 1 week of acclimatization, the body weight of the animals was measured, and they were divided into four groups.

### Experimental design

**Fig. 1:**
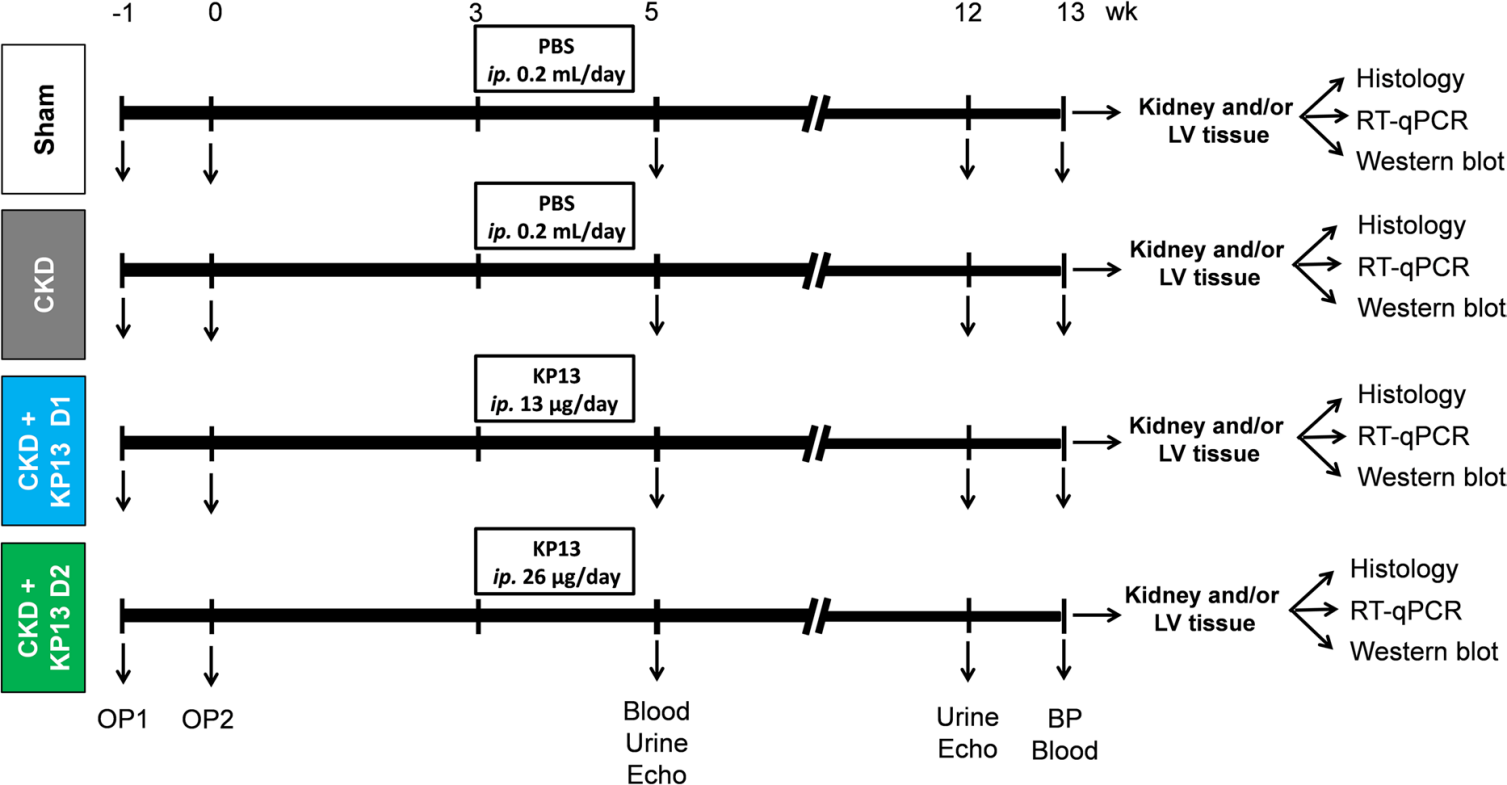
Experimental setup. Blood, blood sampling; BP, blood pressure; CKD, chronic kidney disease group; CKD + KP-13 D1, chronic kidney disease group treated with the lower dose (13 µg/day, dose 1) of the KISS1R agonist kisspeptin-13; CKD + KP-13 D2, chronic kidney disease group treated with the higher dose (26 µg/day, dose 2) of the KISS1R agonist kisspeptin-13; Echo, echocardiography; *ip.*, intraperitoneal; LV, left ventricle; OP, operation; PBS, phosphate-buffered saline; RT-qPCR, real-time quantitative polymerase chain reaction; Sham, sham-operated group; Urine, urine sampling

Out of 32 rats, *n* = 24 received 5/6th nephrectomy to induce experimental CKD, while *n* = 8 sham-operated animals served as controls. After the operations, rats were followed up for 13 weeks (Fig. 1). From the first day of the 3rd follow-up week, rats were treated for 10 days as follows: (I) vehicle-treated (phosphate-buffered saline [PBS], *ip.* 0.2 mL/day) sham-operated group (sham, *n* = 8), (II) vehicle-treated (PBS, *ip.* 0.2 mL/day) CKD group (i.e., CKD-only group, *n* = 8), (III) CKD group treated with a lower dose of KP-13 (*ip.* 13 µg/day [≈8 nmol/day] dissolved in 0.2 mL PBS, CKD + D1, *n* = 8), and (IV) CKD group treated with a higher dose of KP-13 treated (26 µg/day [≈16 nmol/day] dissolved in 0.2 mL PBS, CKD + D2, *n* = 8). The time course and doses of KP-13 were selected based on our preliminary data and previous studies [28, 31–34]. In our study, one animal died in the higher dose of the KP-13-treated CKD group.

At weeks 5 and 12, cardiac morphology and function were assessed by transthoracic echocardiography (Fig. 1). Blood was collected from the saphenous vein at week 5 and from the abdominal aorta at week 13 to measure serum parameters. The animals were placed into metabolic cages for 24 h at weeks 5 and 12 to measure urine creatinine and protein levels (Fig. 1). At week 13, invasive blood pressure measurements were performed in a subgroup of animals (Fig. 1). After the blood pressure measurement, the abdominal cavity was opened to collect 1–1.5 mL of blood from the aorta to measure kidney functional parameters, including serum urea and creatinine levels. To euthanize the rats, sodium pentobarbital was overdosed (Euthasol, 200 mg/kg, *ip.*; Produlab Pharma b.v., Raamsdonksveer, the Netherlands). The hearts, left kidneys, lungs, and tibias were isolated, and the blood was washed out in calcium-free Krebs–Henseleit solution. The hearts and kidneys were weighed, left and right ventricles (LV and RV, respectively) were separated and weighed, the LVs at the ring of the papillae were transversally cut, and a middle cross-sectional ring of the left kidney was cut and fixed in 4% buffered formalin for histological analysis. Other parts of the LVs and kidneys were snap-frozen in liquid nitrogen and stored at − 80 °C until further biochemical measurements and kidney samples were used to measure ACE activity. The development of cardiomyocyte hypertrophy, glomerular and tubular morphology, and cardiac and renal fibrosis in CKD was verified on hematoxylin–eosin (HE) and picrosirius red/fast green (PSFG)-stained sections, respectively (Fig. 1). The expression of selected hypertrophy-, fibrosis-, heart failure (HF)-, inflammation-, and apoptosis-associated markers was measured by RT-qPCR and/or Western blot in tissue samples. Cell culture experiments were performed in human ventricular cardiac fibroblasts (HVCF) as previously described [34, 35].

### Peptide synthesis of KP-13

KP-13 (peptide sequence: H-Leu-Pro-Asn-Tyr-Asn-Trp-Asn-Ser-Phe-Gly-Leu-Arg-Phe-NH_2_, molecular weight: 1626.85 g/mol) was synthesized on a microwave-assisted automated peptide synthesizer (CEM Liberty Blue™, Matthews, NC, USA) in 0.25 mmol scale using Rink Amide 4-methyl-benzhydryl-amine resin (100–200 mesh, 0.52 mmol/g, Merck KGaA, Darmstadt, Germany) and *N*^α^-9-fluorenyl methoxycarbonyl (Fmoc) protected amino acids (fivefold excess). The amino acids (IRIS Biotech GmbH, Marktredwitz, Germany) were activated with *N, N′*-diisopropylcarbodiimide (DIC)/ethyl 2-cyano-2-(hydroxyimino)acetate (Oxyma, Fluorochem Ltd, Hadfield, UK) in situ during the coupling step (90 °C, 2 × 2 min). Removal of the Fmoc groups was done by treating the peptide resin with 10% piperazine (Alfa Aesar GmbH & Co. KG, Karlsruhe, Germany)/10% ethanol in *N*-methyl-2-pyrrolydone (NMP, IRIS Biotech GmbH, Marktredwitz, Germany) mixture (90 °C, 1 min). Wash steps used *N, N*-dimethyl formamide (DMF, Merck KGaA, Darmstadt, Germany).

Peptide was cleaved from the resin by incubating it in a cocktail of 2.5% dithiothreitol (DTT, abcr GmbH, Karlsruhe, Germany), 2.5% triisopropylsilane (TIS, abcr GmbH, Karlsruhe, Germany), 2.5% phenol, 2.5% water in trifluoroacetic acid (TFA) (Merck KGaA, Darmstadt, Germany) for 3 h. The crude peptide was precipitated with cold diethyl ether, filtered, dried, dissolved in an acetic acid/water mixture, and lyophilized. Purification was done on a Shimadzu (Kyoto, Japan) 20AD preparative high-performance liquid chromatography (HPLC) system (Phenomenex Luna C18(2), 10 μm, 100 Å 250 × 21.2 mm column, flow rate: 5 mL/min, wavelength: 210 nm). The purity of the compound was checked by an analytical Agilent (Santa Clara, CA, USA) 1200 HPLC system (Phenomenex Luna C18(2), 10 μm, 100 Å 250 × 4.6 mm column., flow rate: 1 mL/min, wavelength: 210 nm) (Figure S1). Eluent A and B were 0.1% TFA in water and 0.1% TFA/80% acetonitrile (Merck KGaA, Darmstadt, Germany) in water, respectively. The gradient for the analytical HPLC was 40–60% eluent B in eluent A over 20 min (retention time: 8.979 min). The identity of the compound was proved by mass spectrometry (Waters ACQUITY SQ Detector, Milford, MA, USA, calculated and measured molecular weight: 1626.85 and 1626.77, respectively) (Figure S1).

### Subtotal (5/6) nephrectomy model

Sham operation and 5/6th nephrectomy were performed in two phases (Fig. 1), as described previously [34, 36, 37]. Briefly, anesthesia was induced by intraperitoneal injection of sodium pentobarbital (Euthasol; 40 mg/kg; Produlab Pharma b.v., Raamsdonksveer, the Netherlands) before the operations. At the first operation, both poles of the left kidney were excised, approximately at the 1/3rd position. One week after the first operation, the right kidney was freed from the surrounding adipose tissue and the renal capsule, and then it was gently pulled out of the incision. The adrenal gland was gently separated and placed back into the abdominal cavity. During sham operations, only the renal capsules were removed. After the surgeries, the incision was closed with continuous sutures, and povidone-iodine was applied to the skin’s surface. As a postoperative medication, *sc.* 0.3 mg/kg nalbuphine hydrochloride (nalbuphine 10 mg/mL, Teva Pharmaceuticals Ltd., Debrecen, Hungary) was administered for 4 days: twice on the first two and then once on the third and fourth postoperative days. Enrofloxacin antibiotics (Enroxil 75 mg tablets, Krka, Novo Mesto, Slovenia; dissolved in tap water in 3.5 mg/L end concentration) were administered in the drinking water for 4 days after both surgeries.

### Transthoracic echocardiography

Cardiac morphology and function were assessed by transthoracic echocardiography at weeks 5 and 12, as described previously [34, 36, 37] (Fig. 1). Rats were anesthetized with 2% isoflurane (Forane, AESICA, Queenborough Limited Kent, UK). Then, the chest was shaved, and the animal was fixed in a supine position to a heating pad. Two-dimensional, M-mode, Doppler, and tissue Doppler echocardiographic examinations were performed by the criteria of the American Society of Echocardiography using a phased array 5–11 MHz transducer (12S-RS probe, General Electric Medical Systems, New York, NY, USA) connected to a Vivid IQ ultrasound system (General Electric Medical Systems, New York, NY, USA). Data from three consecutive heart cycles were analyzed using the EchoPac Dimension v201 software (General Electric Medical Systems, New York, NY, USA) by an experienced investigator blinded to the group assignment.

In brief, systolic and diastolic wall thickness parameters were obtained from the parasternal short-axis view at the level of the papillary muscles (anterior walls) and the long-axis view at the level of the mitral valve (septal and posterior walls) [38]. The left ventricular diameters were measured using M-mode echocardiography from the short-axis view between the endocardial borders. Fractional shortening (FS) was used as a measure of cardiac contractility (FS = (left ventricular end-diastolic diameter [LVEDD] – left ventricular end-systolic diameter [LVESD])/LVEDD × 100). Functional parameters, including left ventricular end-diastolic volume (LVEDV) and left ventricular end-systolic volume (LVESV), were calculated on four-chamber view images delineating the endocardial borders in diastole and systole. The ejection fraction (EF) was calculated using the formula (LVEDV − LVESV)/LVEDV ∗ 100. Stroke volume (SV) and cardiac output (CO) were calculated using the formula SV = LVEDV-LVESV and CO = SV ∗ heart rate (HR). Diastolic function was assessed using pulsed-wave Doppler across the mitral valve from the apical four-chamber view and tissue Doppler images at the septal mitral annulus. Early (E) mitral flow, septal mitral annulus velocities (e’), and their ratio (E/e’) indicate diastolic function. Heart rate was calculated using pulse-wave Doppler images while measuring transvalvular flow velocity profiles according to the length of three consecutive heart cycles measured between the start points of the E waves.

### Blood pressure measurement

At week 13, a PE50 polyethylene catheter (Cole-Parmer, Vernon Hills, IL, USA) was inserted into the left femoral artery under sodium pentobarbital anesthesia (Euthasol; 40 mg/kg; Produlab Pharma b.v., Raamsdonksveer, the Netherlands) [34, 36, 37] (Fig. 1). Blood pressure measurements were performed between 09:00 and 14:00 h using an SEN-02 pressure transducer (MDE Ltd., Budapest, Hungary) connected to the EXP-HG-1 amplifier (MDE Ltd., Budapest, Hungary) and WS-DA data acquisition system (MDE Ltd., Budapest, Hungary). Data were evaluated by S.P.E.L. Advanced Haemosys software (MDE Ltd., Budapest, Hungary) [34, 36, 37]. The investigators evaluating blood pressure data were blinded to the group assignment.

### Serum and urine metabolite concentrations

At weeks 5 and 12, animals were placed into metabolic cages (Tecniplast Metabolic Cage System, Buguggiate, Italy) for 24 h to measure urine creatinine and protein levels. As described previously, urine creatinine and protein levels were measured by standard laboratory methods [34, 36, 39, 40]. Blood was collected from the saphenous vein at week 5 and from the abdominal aorta at week 13 to measure serum carbamide (urea) and creatinine levels to verify the development of CKD. Urea and creatinine levels in serum were quantified by kinetic UV spectrophotometric method using urease and glutamate dehydrogenase enzymes according to the Jaffe method. The reagents and the platform analyzers were from Roche Diagnostics (Hoffmann-La Roche Ltd., Basel, Switzerland) [39, 40]. Creatinine clearance, an indicator of renal function, was calculated according to the standard formula (urine creatinine concentration [μM] × urine volume for 24 h [mL])/(serum creatinine concentration [μM] × 24 × 60 min). At weeks 5 and 12 or 13, urine creatinine and volume and serum creatinine concentration were measured [39, 40].

### Serum uremic toxin levels

The levels of serum uremic toxins were measured according to previously published methodologies using ultra-high performance liquid chromatography-tandem mass spectrometry (UHPLC-MS/MS) [34, 41, 42]. MRM transition of indoxyl sulfate was 211.9/131.9 using − 50 V as declustering potential and − 25 V as collision energy, retention time: 11.48 min. MRM transition of p-cresyl sulfate was 186.9/107.0 using − 50 V as declustering potential and − 26 V as collision energy, retention time—12.50 min.

### Hematoxylin–eosin, picrosirius red and fast green stainings

Formalin-fixed paraffin-embedded subvalvular areas of the left ventricles and the middle cross-sectional rings of the left kidneys were cut into 5 µm sections and were stained with hematoxylin–eosin (HE) or picrosirius red/fast green (PSFG) as described previously [34, 36, 43, 44]. Histological slides were scanned with a Pannoramic Midi II scanner (3DHistech Ltd, Budapest, Hungary). Digital slide processing was performed in SlideViewer version 2.6 (3DHistech Ltd, Budapest, Hungary; https://www.3dhistech.com/research/software-downloads/, last accessed on 3 January 2023). On the digital HE images, cardiomyocyte cross-sectional areas were measured to verify the development of LVH at the cellular level.

The Biology Image Analysis Software (BIAS 1.0, Single-Cell Technologies Ltd., Szeged, Hungary, https://single-cell-technologies.com/bias/) was used to evaluate HE images [34, 36, 43, 44]. Image pre-processing was followed by deep learning-based cytoplasm segmentation. User-selected objects were forwarded to the feature extraction module configurable to extract properties from the selected cell components. The transverse cardiomyocyte diameter at the nuclear level and cell perimeter were measured in 100 round-shaped and mononucleated cardiomyocytes in the whole digitalized histological slide of the LV tissue block. The BIAS software also calculated cardiomyocyte cross-sectional areas of the same cells. The investigator analyzing the images was blinded to the group assignment.

A pathologist evaluated the histology slides of the kidney (10 × magnification) by QuantCenter (version 2.3) HistoQuant module (3DHistech Ltd, Budapest, Hungary), and the following factors have been examined, graded, and registered, with the use of both HE and PSFG slides: glomerular hypertrophy (0: not present, 1: 1,5 × glomerular size increase present in at least 50% of the glomeruli), tubular dilatation (0: not present, 1: present in less than 50% of tubules, 2: present in more than 50% of tubules), arteriole hyalinosis (0: not present, 1: present), and chronic pyelonephritis (0: not present, 1: present < 50%, 2: present > 50%). Moreover, 10 average glomerular and 10 average tubular diameters have been measured and registered in each case. In the selected anatomical structures, the largest diameters of the opposite basement membranes were registered. The pathologist was blinded to the group assignment.

Cardiac and renal interstitial fibrosis was assessed on PSFG slides with an in-house developed program, as described previously [34, 36, 37]. Briefly, this program determines the proportion of red pixels in whole digitalized LV and kidney slides using two simple color filters. For each Red–Green–Blue (RGB) pixel, the program calculates the pixel color in the Hue-Saturation-Luminance (HSL) color space. The first filter is used for detecting red portions of the image. The second filter excludes any white (empty) or light gray (residual dirt on the slide) pixel from further processing using a simple RGB threshold. In this way, the program groups each pixel into two sets: pixels considered red and pixels considered green but not red, white, or gray. Red pixels in the first set correspond to connective tissue and fibrosis. Green pixels in the second set correspond to cardiac muscle. Dividing the number of elements in the first set by the number of elements in both sets gives the proportion of the connective tissue compartment of the heart area examined.

### Transcription profiling by RT-qPCR in kidney and left ventricular samples

Total RNA was extracted from the kidney and LV samples using the RNeasy Mini Kit (Qiagen, Hilden, Germany) and quantified by NanoDrop spectrophotometer as described previously [36, 44]. Then, 100 µg of total RNA was reverse transcribed using the iScript cDNA Synthesis Kit (Bio-Rad Laboratories Inc., USA). Samples were analyzed in technical duplicates using a 10 µL reaction volume. The initial denaturation step of 3 min at 95 °C was followed by 40 cycles of 15 s 95 °C, 30 s 60 °C, and 40 s 72 °C, using a CFX-96 thermocycler with the accompanying CFX Manager software (Bio-Rad Laboratories Inc., USA) for relative quantification with standard curve analysis using the SQ values. Specific primers (*Bax*: BCL2-associated X apoptosis regulator, #qRnoCED0002625; *Bcl2*: B-Cell CLL/lymphoma 2 apoptosis regulator, #qRnoCED0006419; *Casp7*: apoptosis-related cysteine peptidase, #qRnoCED00051028; *Col1a1*: collagen type 1 alpha 1 chain, #qRnoCED0007857; #qRnoCID0005033; *Ctgf*: connective tissue growth factor, #qRnoCED0001593; *Il6:* interleukin-6, #qRnoCID0053166; *Myh6*: α-myosin heavy chain, #qRnoCID0001766; *Myh7*: β-myosin heavy chain, #qRnoCED0001215; *Nppa*: A-type natriuretic peptide, #qRnoCED0006216; *Nppb*: B-type natriuretic peptide, #qRnoCED0001541; *Tgfb*: transforming growth factor-β, #qRnoCID0009191, *Tnf*: tumor necrosis factor-α, #qRnoCED0009117) and SsoAdvanced Universal SYBR Green Supermix (BioRad Laboratories Inc., USA) were used according to the manufacturer’s instructions. Ribosomal protein lateral stalk subunit P2 (*Rplp2*, forward primer sequence: *agcgccaaagacatcaagaa* and reverse primer sequence: *tcagctcactgatgaccttgtt*) was used as a housekeeping control gene for normalization. In cases of renal kisspeptin-1 (*Kiss1*) and angiotensin-II receptor type 2 (*Agtr2*) expressions, only the absolute Cq values are given (see the detailed method description in the supplementary material).

### Western blot

A standard Western blot technique was used [43, 44] to investigate the expression at the protein level of BAX (20 kDa), CASP7 (35 kDa) with β-actin (45 kDa); BCL-2 (26 kDa), ERK1/2 (42 and 44 kDa), pERK1/2 (42 and 44 kDa), KISS1R (40–140 kDa) applying GAPDH (37 kDa) as a loading control. Left ventricular samples (*n* = 28) were homogenized with an ultrasonicator (UP100H, Hielscher Ultrasonics GmbH, Germany) in Radio-Immunoprecipitation Assay (RIPA) buffer (50 mM Tris–HCl, pH = 8.0), 150 mM NaCl, 0.5% sodium deoxycholate, 5 mM ethylenediamine tetra-acetic acid (EDTA), 0.1% sodium dodecyl sulfate, 1% NP-40; Cell Signaling Technology Inc., USA) supplemented with phenylmethanesulfonyl fluoride (PMSF; Sigma-Aldrich, USA), sodium orthovanadate (Na_3_VO_4_; SigmaAldrich, USA), and sodium fluoride (NaF; Sigma-Aldrich, USA). The crude homogenates were centrifuged at 15,000 × g for 30 min at 4 °C. After quantifying the supernatants’ protein concentrations using the BCA Protein Assay Kit (Pierce Thermo Fisher Scientific Inc., USA), 50 µg of reduced and denaturized protein was loaded in case of BAX and CASP7 and 25 µg in the other cases. Then, sodium dodecyl-sulfate polyacrylamide gel electrophoresis (SDS-PAGE, 50 V, 4 h) was performed with 10% gel, followed by the transfer of proteins onto a nitrocellulose membrane (10% methanol, 35 V, 2 h in case of BCL-2, ERK1/2, pERK1/2, and KISS1R, and 15% methanol, 35 V, 90 min in case of BAX and CASP 7). The efficacy of transfer was checked using Ponceau staining. The membranes were cut vertically and horizontally into parts corresponding to the molecular weights of each protein. Membranes were blocked for 1 h in 5% (w/v) bovine serum albumin (BSA, Sigma-Aldrich, USA) supplemented with Na_3_VO_4_ and NaF and were incubated with primary antibodies in the concentrations of 1:500 against Anti-KISS1R (# AKR001AN0350, Alomone Labs, Israel), and BAX (#14796S, Cell Signaling Technology Inc., USA); 1:1000 against CASP7 (#9492S, Cell Signaling Technology Inc., USA), BCL-2 (#196,495, Abcam PLC, UK), β-actin (#4970S, Cell Signaling Technology Inc., USA), ERK1/2 (#4696S, Cell Signaling Technology Inc., USA), pERK1/2 (#9911 T, Cell Signaling Technology Inc., USA) and 1:5000 against GAPDH (#2118, Cell Signaling Technology Inc., USA) overnight at 4 °C in 5% BSA. Then, the membranes were incubated with IRDye 800CW Goat Anti-Rabbit and/or IRDye 680RD Goat Anti-Mouse secondary antibody (LI-COR Biosciences, USA, in the concentrations of 1:20,000) for 1 h at room temperature in 5% BSA to detect proteins with similar molecular weight on the same membrane where it is applicable. Fluorescent signals were detected by Odyssey CLx machine (LI-COR Biosciences, Lincoln, NE, USA), and digital images were analyzed and evaluated by densitometry with Quantity One Software (Bio-Rad Laboratories Inc., USA). The investigator analyzing the images was blinded to the group assignment.

### ACE activity measurement

ACE activity in kidney tissue samples was measured as previously described [45]. Briefly, tissue samples were weighted, and a proportional amount of 100 mM tris(hydroxymethyl)aminomethane hydrochloride (TRIS) buffer (pH 7.0) was added then homogenized. The tissue homogenates were centrifuged at 15,000 g for 5 min, and the protein concentration of the supernatant was determined by a PierceTM BCA Protein Assay Kit (Thermo Fisher Scientific) using a TECAN (SparkControl Magellan V2.2) plate reader. ACE activity was determined with an artificial substrate (Abz-FRK(Dnp)P-OH (synthesized by Peptide 2.0, Chantilly, VA, USA) in a reaction mixture containing 6 µl of 1 mg/mL tissue homogenates in 35-fold dilution in 100 mM TRIS buffer, 50 mM NaCl, 10 µM ZnCl_2_. Measurements were performed in 96-well plates (Greiner-Bio One) at 37 °C. The fluorescence intensity change was detected by a TECAN (SparkControl Magellan V2.2) plate reader (excitation, 340 nm; emission, 405 nm). The changes in fluorescence intensity were detected in kinetic loops, at 1-min intervals for at least 30 min, and the intensity values were plotted as a function of reaction time. The fluorescence intensity values were fitted by a linear regression (GraphPad Software, San Diego, CA, USA), and the fit with the data was accepted only when r^2^ was > 0.9. ACE activity was calculated by the following equation: activity = (S/k) × D/P, where S is the rate of the increase in fluorescence intensity (1/min), k is the change in fluorescence intensity during the complete cleavage of 1 pmol Abz-FRK(Dnp)P-OH substrate, D is the dilution of the sample, and P is the mg/mL protein concentration; 1 unit (U) means 1 pmol substrate cleavage in 1 min by 1 mg of protein.

### Cell culture experiments and RT-qPCR

Human ventricular cardiac fibroblasts (HVCFs, cryopreserved ampules of normal human ventricular cardiac fibroblasts containing ≥ 500,000 cells, #CC-2904, Lonza, Basel, Switzerland, https://bioscience.lonza.com/lonza_bs/CH/en/Primary-and-Stem-Cells/p/000000000000197234/NHCF-V-%E2%80%93-Human-Ventricular-Cardiac-Fibroblasts) were cultured in a fibroblast basal medium supplemented with 0.1% insulin, 0.1% fibroblast growth factor, 0.1% GA-1000, 1% pen-strep, and 10% FBS (all Lonza, Basel, Switzerland) as previously described [35]. Cultures were washed with HEPES buffered saline (Lonza, Basel, Switzerland) when indicated and split at a confluency level of 70% [35]. Cells were treated as follows: (I) without treatment—control; (II) 20 ng/mL transforming growth factor-beta (TGF-β, R&D systems, Minneapolis, Minnesota, USA); (III) 100 nmol/L KP-13; and (IV) 20 ng/mL TGF-β with 100 nmol/L KP-13 for 24 h.

Total RNA was extracted from HVCFs using the RNeasy Plus Micro Kit (#74,037, Qiagen, Hilden, Germany) and quantified by Tecan spectrophotometer Tecan Group Ltd (Tecan Group Ltd, Männedorf, Switzerland). cDNA was prepared using the QuantiTect reverse transcription kit (Qiagen, Hilden, Germany). Samples were analyzed in technical duplicates using a 20 µL reaction volume. The initial denaturation step of 3 min at 95 °C was followed by 40 cycles of 15 s 95 °C, 30 s 60 °C, and 30 s 72 °C, using a CFX Opus thermocycler with the accompanying software (Bio-Rad Laboratories Inc., USA) for Ct value analysis, and gene expressions of *Col1*, matrix metalloprotease-9 (*Mmp9*), and α-smooth muscle actin (*Acta2*) were determined relative to glyceraldehyde-3-phosphate dehydrogenase (*Gapdh*) and hypoxanthine–guanine phosphoribosyltransferase (*Hgprt*) expressions using RT-qPCR and 2^−ΔΔCq^ method. The primer sequences are described in Table 1.

**Table 1:** Primer sequences used in RT-qPCR experiments in HVCFs

Gene symbol (species)	Forward primer sequence	Reverse primer sequence
*Acta2* (human)	CCA GAG CCA TTG TCA CAC AC	CAG CCA AGC ACT GTC AGG
*Col1a1* (human)	AGT CGA GGG CCA AGA CGA AG	ACA ACA CCT TGC CGT TGT CG
*Mmp9* (human)	GAC GAC CGG TTT GGC TTC TG	GAG CTT GTC CCG GTC GTA GT
*Hgprt* (human)	TGA CAC TGG CAA AAC AAT GCA	AAG CTT GCG ACC TTG ACC AT
*Gapdh* (human)	TCC TGT TCG ACA GTC AGC CG	CCC CAT GGT GTC TGA GCG AT

### Statistical analysis

Statistical analyses were performed using Sigmaplot 14.0 software for Windows (Systat Software Inc., San Jose, CA, USA). All values are presented as mean ± SEM; *p* < 0.05 was accepted as a statistically significant difference. One-way ANOVA was used to determine the statistical significance between all measured parameters within each time point. Two-way repeated-measures ANOVA with Holm-Sidak’s *post-hoc* test was used to determine the effects of CKD and the treatments on serum, urine, and echocardiographic parameters between week 5 and endpoint follow-up data. For Western blot results of KISS1R, ERK1/2, and pERK1/2, an unpaired *t*-test was also used to investigate the statistical significance between CKD-only vs. sham-operated groups or KP-13-treated CKD vs. CKD-only groups. Fisher’s test was carried out for renal histology scoring using R software (version 4.2.3 for Windows) to compare outcome measures between treatment groups.

## Results

### Effects of KP-13 on serum urea levels in CKD

**Fig. 2:**
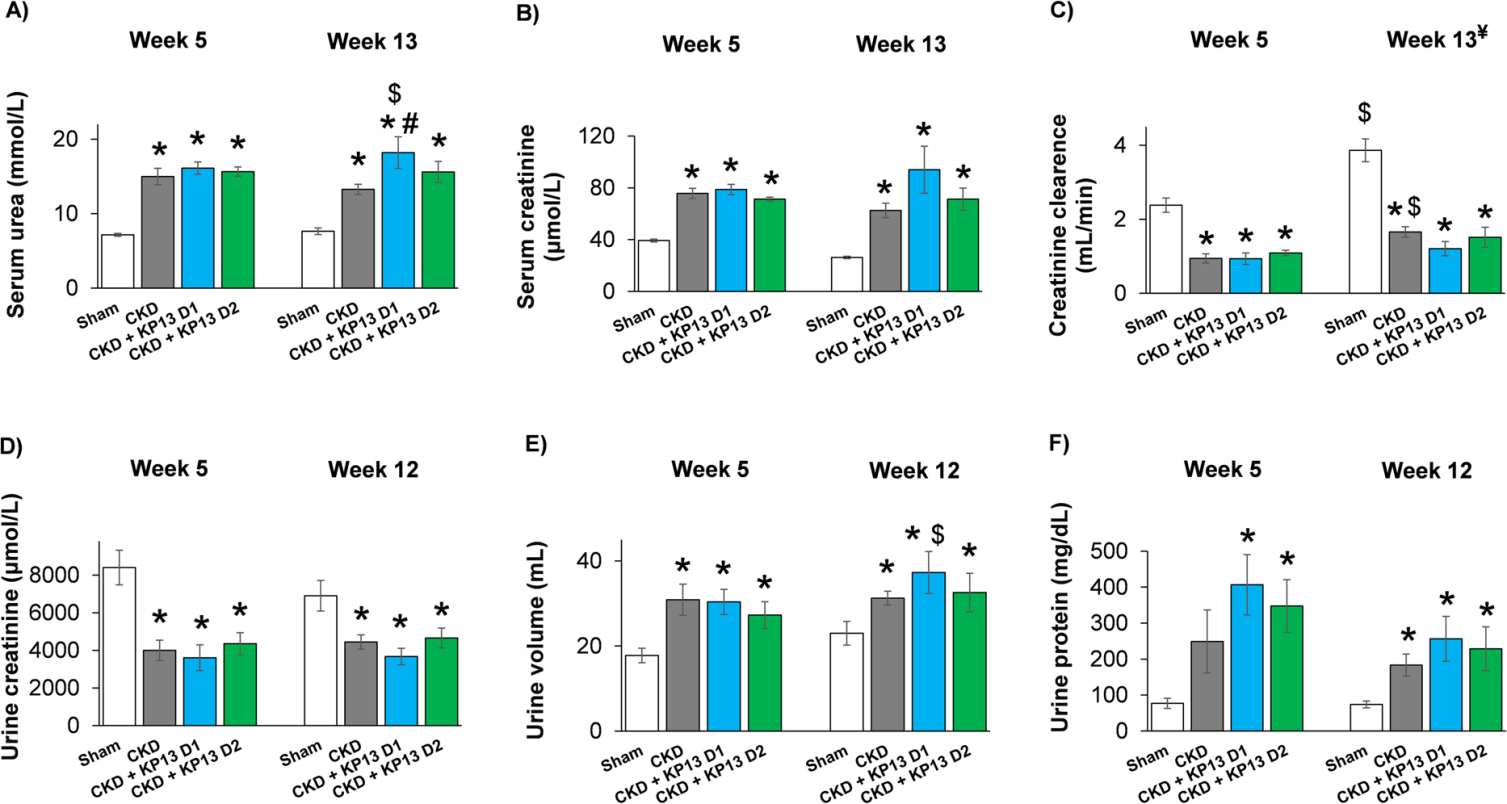
The effects of the KISS1R agonist kisspeptin-13 on serum and urine parameters of CKD in 5/6 nephrectomized rats. **A** Serum urea concentration, **B** serum creatinine concentration, **C** creatinine clearance, **D** urine creatinine concentration, **E** urine volume, and **F** urine protein concentration at week 5 and the endpoint. Values are presented as mean ± SEM, *n* = 7–8. *p < 0.05 vs. Sham, and ^#^*p* < 0.05 vs. CKD (one-way ANOVA, Holm-Sidak post hoc test). ^$^*p* < 0.05 vs. week 5 values in the same group (repeated measures two-way ANOVA, Holm-Sidak post hoc test). Creatinine clearance was calculated according to the standard formula (urine creatinine concentration [µM] × urine volume for 24 h [mL])/(serum creatinine concentration [µM] × 24 × 60 min). (*p*-value of repeated measure two-way ANOVA for interaction in creatinine clearance: 0.012). ^¥^At the endpoint, urine volume and creatinine concentration were measured at week 12 and serum creatinine concentration at week 13. Sham, sham-operated group; CKD, chronic kidney disease group; CKD + KP-13 D1, chronic kidney disease group treated with the lower dose (13 µg/day, dose 1) of the KISS1R agonist kisspeptin-13; CKD + KP-13 D2, chronic kidney disease group treated with the higher dose (26 µg/day, dose 2) of the KISS1R agonist kisspeptin-13

At the 5th and 12th–13th follow-up weeks, serum and urine parameters were determined to investigate the effects of KP-13 on the laboratory signs of CKD (Figs. 1 and 2). At week 5 and the endpoint, all CKD groups showed significantly higher serum urea and creatinine concentrations, urine volume, and decreased creatinine clearance and urine creatinine level compared to the sham-operated group (*p < 0.05), indicating impaired renal function from week 5 onward (Fig. 2A, B, C, D, and E). In the CKD-only group, the urine protein levels were tendentiously (*p* = 0.058) increased at week 5 and significantly elevated at the endpoint compared to the sham-operated group (*p < 0.05), indicating a diminished glomerular function (Fig. 2F). In both KP-13-treated CKD groups, the urine protein levels were markedly elevated at week 5 and the endpoint compared to the sham-operated group (*p < 0.05) (Fig. 2F). At the endpoint, there was a significant increase in the serum urea level (^#^*p* < 0.05) and an increasing tendency in the serum creatinine level (*p* = 0.09) as well as a decreasing tendency in the creatinine clearance (*p* = 0.08) in the lower dose of KP-13-treated CKD group, compared to the CKD-only group (2A-2C). In contrast, there were no significant differences in the measured serum and urine parameters in the higher dose of the KP-13-treated CKD group compared to the CKD-only group (Fig. 2A-F). The creatinine clearance was significantly increased in the CKD-only and sham-operated groups at the endpoint compared to the week 5 values in the same group (^$^*p* < 0.05), which could be attributed to the growth of the animals (Fig. 2C). At the endpoint, the serum urea level and the urine volume were significantly elevated in the lower dose of the KP-13-treated CKD group compared to the week 5 values (^$^*p* < 0.05) (Fig. 2A and E).

### KP-13 exacerbates glomerular hypertrophy and tubular dilatation in CKD

**Fig. 3:**
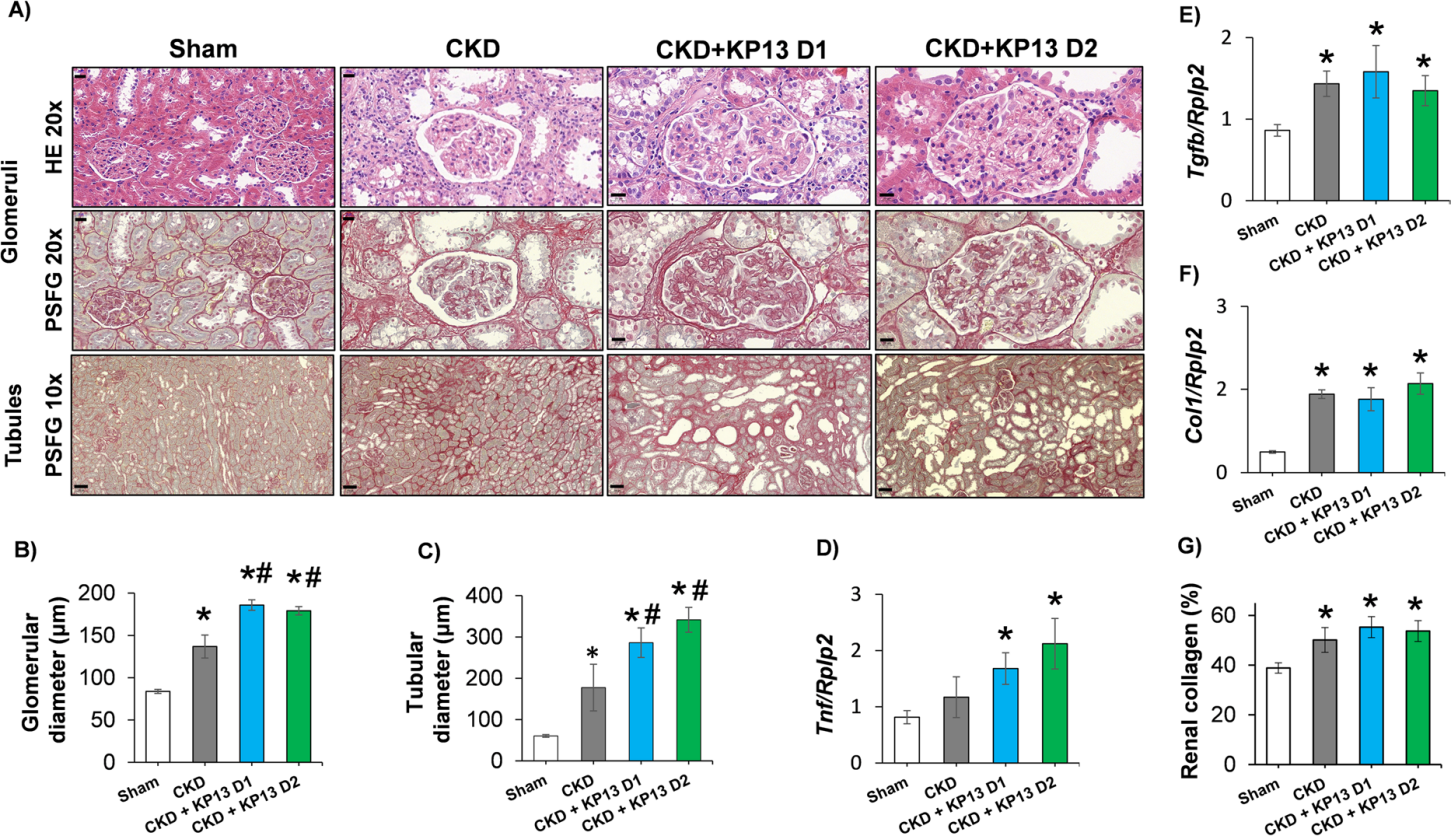
The effects of the KISS1R agonist kisspeptin-13 on renal morphology and inflammatory and fibrotic markers in CKD at the endpoint. **A** Representative hematoxylin–eosin (HE, × 20 magnification)- and picrosirius red/fast green (PSFG)-stained slides (× 10 and × 20 magnifications) 10 and 20 magnifications) of glomeruli and tubules; **B** glomerular diameter; and **C** tubular diameter. Renal expression of **D** tumor necrosis factor-α (*Tnf*), **E** transforming growth factor-β (*Tgfb*); and **F** collagen-1 (*Col1*). **G** Renal collagen content. Values are presented as mean ± SEM, *n* = 7–8. *p < 0.05 vs. Sham, #*p* < 0.05 vs. CKD (one-way ANOVA, Holm-Sidak post hoc test). Sham, sham-operated group; CKD, chronic kidney disease group; CKD + KP-13 D1, chronic kidney disease group treated with the lower dose (13 µg/day, dose 1) of the KISS1R agonist kisspeptin-13; CKD + KP-13 D2, chronic kidney disease group treated with the higher dose (26 µg/day, dose 2) of the KISS1R agonist kisspeptin-13. Gene expressions are normalized to the ribosomal protein lateral stalk subunit P2 (*Rplp2*) housekeeping gene expression

To assess the effects of KP-13 on renal morphology in CKD, glomerular and tubular diameters and renal fibrosis were measured on HE- and PSFG-stained slides (Fig. 3A). Glomerular diameters were significantly increased in all CKD groups compared to the sham-operated group (*p < 0.05), indicating the development of glomerular hypertrophy (Fig. 3B). Similarly, tubular diameters were also increased in all CKD groups compared to the sham-operated group, indicating the development of tubular dilatation (Fig. 3C). In both KP-13-treated CKD groups, the glomerular and tubular diameters were markedly increased compared to the CKD-only group (^#^*p* < 0.05). Additionally, Fisher’s test revealed a statistically significant association between KP-13 treatment in CKD and arteriola hyalinosis (*p* = 0.0001744), as well as chronic pyelonephritis (*P* = 0.00006935) (Figure S2). Moreover, the inflammatory marker, tumor necrosis factor-α (*Tnf*) was significantly overexpressed in the KP-13-treated CKD groups compared to the sham-operated group (*p < 0.05) (Fig. 3A, B, C, and D). KP-13 administration failed to increase further the significant overexpression of the fibrotic markers transforming growth factor-β (*Tgfb*) and collagen-1 (*Col1*) compared to the CKD-only group (Fig. 3E and F). Indeed, collagen content was significantly increased in all CKD groups compared to the sham-operated group (*p < 0.05) (Fig. 3A and G).

### KP-13 reduces LV wall thicknesses and worsens diastolic dysfunction in CKD

**Fig. 4:**
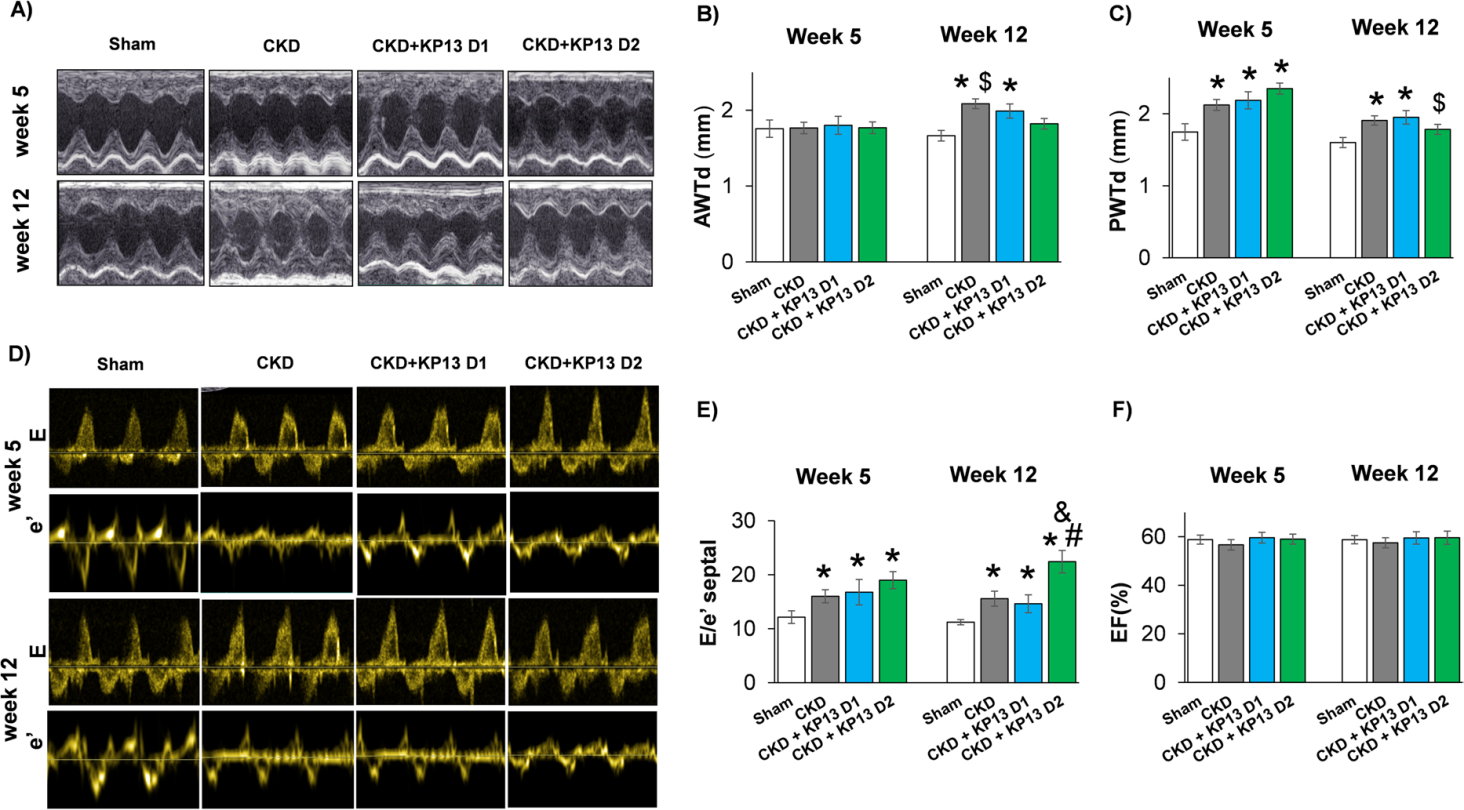
The effects of the KISS1R agonist kisspeptin-13 on the echocardiographic parameters of uremic cardiomyopathy. **A** Representative M-mode images, **B** diastolic anterior wall thickness (AWTs), **C** diastolic posterior wall thickness (PWTd), **D** representative pulse wave and tissue Doppler images of mitral valve early flow velocity and **E** septal mitral annulus velocities (e’), **E** E/e’ ratio, **F** ejection fraction (EF). Values are presented as mean ± SEM, *n* = 7–8. * *p* < 0.05 vs. Sham, ^#^*p* < 0.05 vs. CKD, ^&^*p* < 0.05 vs. CKD + KP-13 D1 (one-way ANOVA, Holm-Sidak post hoc test). ^$^*p* < 0.05 vs. week 5 values in the same group (repeated measures two-way ANOVA, Holm-Sidak post hoc test). Sham, sham-operated group; CKD, chronic kidney disease group; CKD + KP-13 D1, chronic kidney disease group treated with the lower dose (13 µg/day, dose 1) of the KISS1R agonist kisspeptin-13; CKD + KP-13 D2, chronic kidney disease group treated with the higher dose (26 µg/day, dose 2) of the KISS1R agonist kisspeptin-13

At week 5, the posterior walls were significantly thicker, accompanied by markedly reduced left ventricular end-systolic diameters (LVESD), mitral annulus velocities (e’), and significantly increased fractional shortening (FS) and E/e’ ratio in all CKD groups compared to the sham-operated group (*p < 0.05), indicating that LVH and diastolic dysfunction started to develop in an early phase of CKD (Fig. 4, Table 2). It should be mentioned that the systolic anterior and septal wall thicknesses were significantly increased in both KP-13-treated CKD groups compared to the sham-operated group at week 5 (*p < 0.05) (Table 2).

**Table 2:** The Effects of the KISS1R agonist kisspeptin-13 on the echocardiographic parameters of uremic cardiomyopathy at weeks 5 and 12

Parameter (unit)	Week 5				Week 12			
	Sham	CKD	CKD + KP-13 D1	CKD + KP-13 D2	Sham	CKD	CKD + KP-13 D1	CKD + KP-13 D2
AWTs (mm)	3.03 ± 0.14	3.41 ± 0.14	3.52 ± 0.13*	3.5 ± 0.11 *	3.08 ± 0.09	3.56 ± 0.14*	3.54 ± 0.1*	3.41 ± 0.16*
PWTs (mm)	3.12 ± 0.10	3.46 ± 0.11*	3.56 ± 0.11*	3.61 ± 0.12 *	2.77 ± 0.12	3.45 ± 0.15*	3.28 ± 0.17*^$^	3.37 ± 0.17*^$^
SWTs (mm)	3.12 ± 0.14	3.56 ± 0.18	3.98 ± 0.14*	3.92 ± 0.19 *	3.45 ± 0.13	3.64 ± 0.16	3.64 ± 0.1	3.87 ± 0.18
SWTd (mm)	1.7 ± 0.09	1.91 ± 0.09	2.06 ± 0.11	1.88 ± 0.19	1.89 ± 0.15	2.15 ± 0.12	1.96 ± 0.12	1.94 ± 0.16
LVESD (mm)	3.46 ± 0.17	2.16 ± 0.28*	2.55 ± 0.15*	2.13 ± 0.30*	3.36 ± 0.14	2.81 ± 0.26*	2.66 ± 0.23*	2.94 ± 0.35^#^
LVEDD (mm)	6.75 ± 0.21	6.35 ± 0.26	6.60 ± 0.29	6.23 ± 0.25*	7.25 ± 0.32	6.55 ± 0.20*	6.54 ± 0.30	6.58 ± 0.25
FS (%)	51 ± 3	63 ± 4*	60 ± 2 *	64 ± 4 *	54 ± 2	60 ± 3	59 ± 2	58 ± 5
LVESV (µL)	81 ± 7	73 ± 8	75 ± 9	66 ± 12	104 ± 6	85 ± 7*	70 ± 10*	73 ± 11*
LVEDV (µL)	194 ± 12	168 ± 17	187 ± 20	159 ± 24	255 ± 16^$^	199 ± 13*	170 ± 16*	176 ± 18*
SV (µL)	113 ± 6	95 ± 10	112 ± 14	92 ± 13	151 ± 12^$^	114 ± 8*	100 ± 9*	103 ± 9*
HR (BPM)	408 ± 12	396 ± 9	390 ± 8	415 ± 10	406 ± 10	380 ± 15	377 ± 12	409 ± 8
CO (mL/min)	47 ± 3	37 ± 4	43 ± 5	39 ± 6	61 ± 5*^$^	43 ± 4*	38 ± 3*	42 ± 4*
E (m/s)	0.89 ± 0.04	0.91 ± 0.05	0.93 ± 0.05	0.98 ± 0.05	0.96 ± 0.04	0.97 ± 0.05	0.98 ± 0.05	0.98 ± 0.05
e’ (m/s)	0.077 ± 0.005	0.056 ± 0.006*	0.059 ± 0.004*	0.053 ± 0.004*	0.086 ± 0.002	0.065 ± 0.006 *	0.072 ± 0.005 *	0.046 ± 0.005*^#^

Values are presented as mean ± SEM, *n* = 7–8. *p < 0.05 vs. sham, ^#^*p* < 0.05 vs. CKD (one-way ANOVA, Holm-Sidak post hoc test), ^$^*p* < 0.05 vs. week 5 value in the same group (repeated measures two-way ANOVA, Holm-Sidak post hoc test). *AWT* anterior wall thickness, *CO* cardiac output, *d* diastolic, *E-velocity* early ventricular filling velocity, *e’-velocity* diastolic septal mitral annulus velocity. *FS* fractional shortening, *HR* heart rate, *LVEDD* left ventricular end-diastolic diameter, *LVESD* left ventricular end-systolic diameter, *s* systolic, *SWT* septal wall thickness, *SV* stroke volume, *Sham* sham-operated group, *CKD* chronic kidney disease group, *CKD* + *KP-13 D1* chronic kidney disease group treated with the lower dose (13 µg/day, dose 1) of KISS1R agonist kisspeptin 13, *CKD* + *KP-13 D2* chronic kidney disease group treated with the higher dose (26 µg/day, dose 2) of KISS1R agonist kisspeptin 13

At week 12, in the CKD-only group, the anterior and posterior walls were significantly thicker, and the LVEDD and LVESD were significantly decreased compared to the sham-operated group (*p < 0.05), indicating the development of LVH (Fig. 4A, B, and C, Table 2). In the lower dose of the KP-13-treated CKD group, the anterior and posterior walls remained significantly thicker, and the LVESD was markedly reduced compared to the sham-operated group (*p < 0.05) (Fig. 4A, B, and C, Table 2). Interestingly, in the higher dose of the KP-13-treated CKD group, only the systolic anterior and posterior wall thicknesses remained significantly increased compared to the sham-operated group (*p < 0.05) (Table 2). In contrast, there were no significant differences in the diastolic anterior and posterior walls, as well as LVESD and LVEDD, between the sham-operated and the higher dose of the KP-13-treated CKD groups (Fig. 4A, B, and C, Table 2). The FS used as a contractile function parameter was preserved in all CKD groups (Table 2). Notably, the LVESD and the E/e’ were significantly increased, and the e’ was markedly decreased in the higher dose of the KP-13-treated CKD group compared to the CKD-only group (^#^*p* < 0.05) (Table 2, Fig. 4D and E). Moreover, the E/e’ was significantly higher in the higher dose of the KP-13-treated CKD group compared to the lower dose of the KP-13-treated CKD group at week 12 (^&^*p* < 0.05) (Fig. 4D and E).

When comparing the week 12 values to those of week 5, the diastolic anterior wall thickness was significantly increased in the CKD-only group (^$^*p* < 0.05) (Fig. 4A and B). In contrast, the systolic posterior wall was significantly thinner at week 12 compared to the week 5 value in the KP-13-treated CKD groups (^$^*p* < 0.05) (Table 2). In the higher dose of the KP-13-treated CKD group, the diastolic posterior wall was also markedly thinner compared to the week 5 value (^$^*p* < 0.05) (Fig. 4C).

At week 5, there was no significant difference in the left ventricular end-systolic and end-diastolic volumes (LVESV and LVEDV, respectively) and stroke volume (SV), heart rate, and cardiac output (CO) among the groups (Table 2). However, it should be mentioned that the CO tended to decrease in the CKD-only compared to the sham-operated group (*p* = 0.052) (Table 2). At week 12, the LVESV, LVEDV, SV, and CO were significantly reduced in all CKD groups compared to the sham-operated group (*p < 0.05) (Table 2). There were no significant differences in the global pump function represented by the ejection fraction (EF) among the groups at weeks 5 or 12 weeks (Fig. 4F).

### KP-13 reduces LV weight in CKD

In consonance with the echocardiography and renal histology results, the heart, LV, and kidney weights, as well as the LV to tibia length ratio, were significantly elevated in the CKD-only group compared to the sham-operated group (*p < 0.05), which are the macroscopic signs of LV as well as renal hypertrophy (Table 3). The lung weight tended to increase in the CKD-only group compared to the sham-operated group, indicating the presence of mild pulmonary edema in CKD (Table 3). In the lower dose of the KP-13-treated CKD group, heart and LV weight, as well as LV weight to tibia length ratio, remained markedly higher compared to the sham-operated group (*p < 0.05), indicating the presence of LVH (Table 3). In contrast, these parameters were not significantly different between the higher dose of KP-13-treated CKD and sham-operated groups, which is consistent with the echocardiographic findings (Table 3). Kidney weights only tended to increase (*p* = 0.07 and *p* = 0.097, respectively) in both KP-13-treated CKD groups compared to the sham-operated group (Table 3). The lung weight was significantly decreased in both KP-13-treated CKD groups compared to the CKD-only group (Table 3). Notably, the body weight was not significantly different between the CKD-only and sham-operated groups; however, it was markedly lower in the KP-13-treated CKD groups compared to the sham-operated group (*p < 0.05) (Table 3).

**Table 3:** The effects of the KISS1R agonist kisspeptin-13 on the body weight, tibia length, and organ weights in CKD at week 13

Parameter (unit)	Sham	CKD	CKD + KP-13 D1	CKD + KP-13 D2
Body weight (g)	443 ± 9	443 ± 19	400 ± 11*	403 ± 10*
Tibia length (cm)	4.21 ± 0.04	4.19 ± 0.06	4.19 ± 0.04	4.17 ± 0.03
Heart weight (mg)	1216 ± 29	1328 ± 41*	1510 ± 98*	1359 ± 94
LV weight (mg)	707 ± 36	852 ± 43*	841 ± 46*	756 ± 32
LV weight/tibia length (mg/cm)	167 ± 7	205 ± 12*	200 ± 12*	181 ± 7
RV weight (mg)	194 ± 8	199 ± 12	208 ± 15	210 ± 18
Lung weight (mg)	1477 ± 52	1635 ± 81	1385 ± 29^#^	1407 ± 29^#^
Kidney weight (mg)	1186 ± 44	1487 ± 126*	1379 ± 106	1343 ± 79

Values are presented as mean ± SEM, *p < 0.05 vs. sham, ^#^*p* < 0.05 vs. CKD, *n* = 7–8 (one-way ANOVA, Holm-Sidak post hoc test). *CKD* chronic kidney disease group, *CKD* + *KP-13 D1* chronic kidney disease group treated with the lower dose (13 µg/day, dose 1) of the KISS1R agonist kisspeptin-13, *CKD* + *KP-13 D2* chronic kidney disease group treated with the higher dose (26 µg/day, Dose 2) of the KISS1R agonist kisspeptin-13, *LV* left ventricular, *RV* right ventricular, *Sham* sham-operated group

### KP-13 reduces cardiomyocyte cross-sectional areas in CKD

**Fig. 5:**
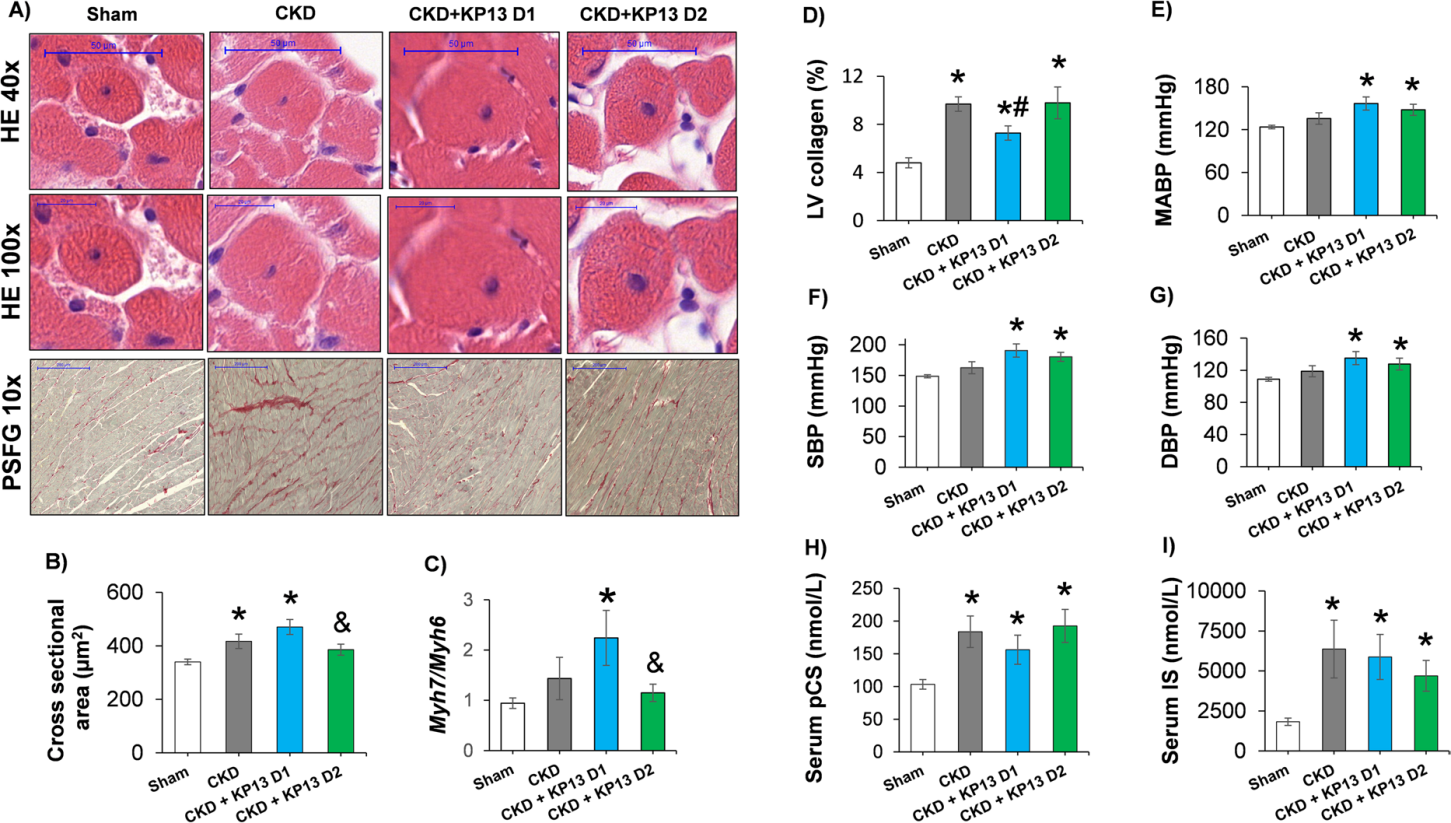
The effects of the KISS1R agonist kisspeptin-13 on cardiomyocyte hypertrophy, interstitial fibrosis, and several risk factors and molecular markers in CKD at week 13. **A** Representative hematoxylin–eosin (HE)-stained slides at × 40 and × 100 magnifications and representative picrosirius red and fast green (PSFG)-stained slides at × 10 magnification. **B** Cardiomyocyte cross-sectional areas, **C** left ventricular (LV) collagen content. Serum **D** para-cresyl sulfate (pCS), and **E** indoxyl sulfate levels. **F** Systolic blood pressure (SBP), **G** diastolic blood pressure (DBP), **H** mean arterial blood pressure, **I** β-myosin heavy chain (Myh7)/α-myosin heavy chain (*Myh6*) ratio. On the digital HE images, cardiomyocyte cross-sectional areas were measured in 100 selected cardiomyocytes on left ventricular sections cut on the same plane. Values are presented as mean ± SEM, *p < 0.05 vs. sham, ^#^*p* < 0.05 vs. CKD, ^&^*p* < 0.05 vs. CKD + KP-13 D1 (*n* = 7–8, one-way ANOVA, Holm-Sidak post hoc test). Sham, sham-operated group; CKD, chronic kidney disease group; CKD + KP-13 D1, chronic kidney disease group treated with the lower dose (13 µg/day, dose 1) of the KISS1R agonist kisspeptin-13; CKD + KP-13 D2, chronic kidney disease group treated with the higher dose (26 µg/day, dose 2) of the KISS1R agonist kisspeptin-13

Cross-sectional areas of 100 cardiomyocytes/sample were measured on HE-stained histological slides, and collagen content was measured on PSFG-stained slides to investigate the effects of KP-13 on the development of LVH and fibrosis in CKD, respectively (Fig. 5A). Cardiomyocytes showed significantly enlarged cross-sectional areas in the CKD-only and the lower dose of the KP-13-treated CKD group compared to the sham-operated group (*p < 0.05), confirming the development of LVH at the cellular level at week 13 (Fig. 5A and B). In contrast, the cardiomyocyte cross-sectional areas were not significantly different between the higher dose of the KP-13-treated CKD and sham-operated groups, validating the echocardiographic and macroscopic findings (Fig. 5A and B). Moreover, the cardiomyocyte cross-sectional areas were significantly lower in the higher dose of the KP-13-treated CKD group compared to the lower dose of the KP-13-treated CKD group (^&^*p* < 0.05) (Fig. 5A and B).

To strengthen our results at the molecular level, the expression of cardiac hypertrophy markers was measured by RT-qPCR. The increased ratio of the fetal myosin heavy chain β-isoform to the adult α-isoform (β-MHC to α-MHC ratio) is thought to be an indicator of the fetal gene reprogramming in LVH in response to tissue hypoxia [46]. The LV gene expression changes in the β-myosin heavy chain (*Myh7*) to α-myosin heavy chain (*Myh6*) ratios were similar to the findings on the cardiomyocyte cross-sectional areas (Fig. 5B and C). However, the LV *Myh7/Myh6* failed to change significantly in the CKD-only group compared to the sham-operated group at week 13 (Fig. 5C). Significant interstitial fibrosis was found in all CKD groups compared to the sham-operated group (*p < 0.05) (Fig. 5A and D), and this was markedly suppressed in the lower dose of the KP-13-treated CKD group compared to the CKD-only group (^#^*p* < 0.05) (Fig. 5A and D).

### KP-13 increases blood pressure in CKD

Hypertension is a well-known complication and an independent risk factor for developing renal and cardiac hypertrophy and fibrosis in CKD [2, 11]. In our present study, systolic, diastolic, and mean arterial blood pressures showed only increasing tendencies (*p* = 0.091, *p* = 0.102, and *p* = 0,083, respectively) in the CKD-only group compared to the sham-operated group (Fig. 5E, F, and G). In contrast, both KP-13-tretaed CKD groups presented significant systolic, diastolic, and mean arterial blood pressure elevation at similar degrees compared to the sham-operated group (*p < 0.05) (Fig. 5E, F, and G). To further characterize the potential underlying causes of hypertension, hypertrophy, and fibrosis development in CKD, we measured the renal expressions of *Kiss1* and *Agtr2* genes as well as ACE activity and the serum levels of para-cresyl sulfate and indoxyl sulfate [47, 48]. The renal *Kiss1* and *Agtr2* expressions and ACE activity were not significantly different between the groups (Figure S3). However, the renal ACE tended to decrease tendency in the CKD-only group compared to the sham-operated group (*p* = 0.124) (Figure S3). Notably, the renal ACE activity was tendentiously increased in the lower dose of the KP-13-treated CKD group compared to the CKD-only group (*p* = 0.100) (Figure S3). Serum levels of both uremic toxins were significantly increased in all CKD groups compared to the sham-operated group (*p < 0.05), independently of KP-13 administration (Fig. 5H and I).

### Effect of KP13 on LV ANP expression in CKD

**Fig. 6:**
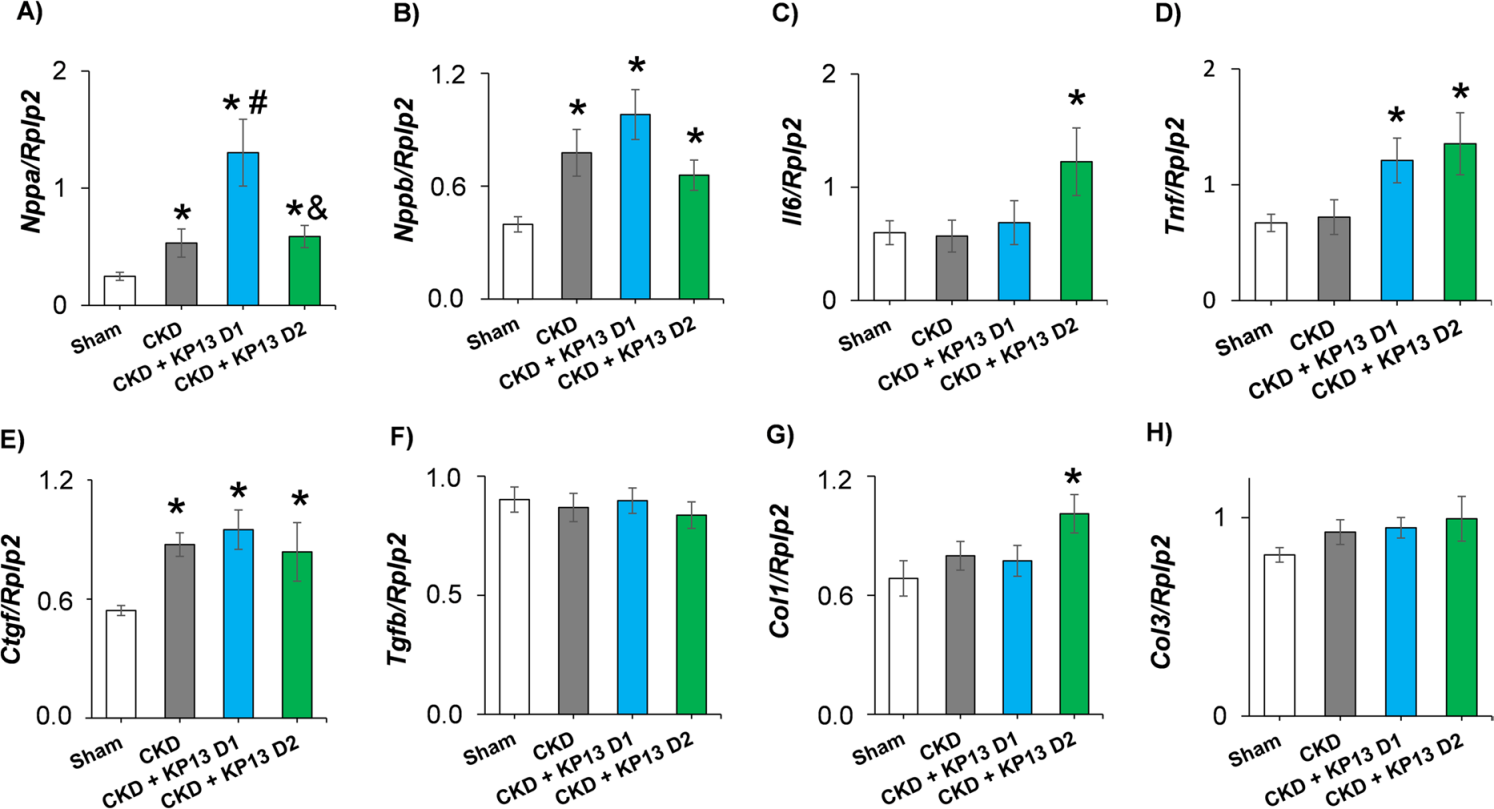
The effects of the KISS1R agonist kisspeptin-13 on the left ventricular expression of genes associated with heart failure, inflammation, and fibrosis. Relative gene expression of **A** A-type natriuretic peptide (*Nppa*), **B** B-type natriuretic peptide (*Nppb*), **C** interleukin-6 (*Il6*), **D** tumor necrosis factor-α (*Tnf*), **E** connective tissue growth factor (*Ctgf*), **F** transforming growth factor-β (*Tgfb*), **G** collagen-1 (*Col1*), and **H** collagen-3 (*Col3*) normalized to the ribosomal protein lateral stalk subunit P2 (*Rplp2*) gene expression. Values are presented as mean ± SEM (*n* = 7–8). *p < 0.05 vs. sham, ^#^*p* < 0.05 vs. CKD, ^&^*p* < 0.05 vs. CKD + KP-13 D1 (one-way ANOVA, Holm-Sidak post hoc test). Sham, sham-operated group; CKD, chronic kidney disease group; CKD + KP-13 D1, chronic kidney disease group treated with the lower dose (13 µg/day, dose 1) of the KISS1R agonist kisspeptin-13; CKD + KP-13 D2, chronic kidney disease group treated with the higher dose (26 µg/day, dose 2) of the KISS1R agonist kisspeptin-13

A-type and B-type natriuretic peptides are natriuretic, diuretic, and vasodilating hormones secreted from the atria and ventricles in response to mechanical stretch-related forces [49]. Indeed, LV expressions of both natriuretic peptide genes (*Nppa* and *Nppb*, respectively) were significantly increased in all CKD groups compared to the sham-operated group (*p < 0.05) (Fig. 6A and B). Interestingly, the expression of the *Nppa* was markedly elevated in the lower dose of the KP13-treated group compared to the CKD-only group (^#^*p* < 0.05) (Fig. 6A). In the higher dose of the KP-13-treated CKD group, the *Nppa* overexpression was markedly reduced compared to the lower dose of the KP-13-treated CKD group (^&^*p* < 0.05) (Fig. 6A). The overexpression of *Nppb* showed a decreasing tendency (*p* = 0.081) in the higher dose of the KP-13-treated CKD group compared to the lower dose of the KP-13-treated group (Fig. 6B).

### Effects of KP13 on LV expression of Il6, Tnf, and Col1 in CKD

To investigate the molecular mechanisms behind the observed cardiac interstitial fibrosis, we evaluated the LV expression of inflammatory and fibrosis markers using RT-qPCR (Figs. 6C, D, E, F, G, and H). Among the evaluated inflammatory markers, interleukin-6 (*Il6*) was significantly overexpressed in the higher dose of KP13-treated CKD group, and tumor necrosis factor-α (*Tnf*) was overexpressed in both KP13-treated CKD groups compared to the sham-operated group (*p < 0.05) (Fig. 6C and D). In our present study, *Ctgf* was markedly overexpressed in all CKD groups, and *Col1* was significantly overexpressed only in the higher dose of the KP-13-treated group compared to the sham-operated group (*p < 0.05). There was no significant difference in the *Tgfb* and *Col3* expressions among the groups (Fig. 6E, F, G, and H).

To further investigate the potential anti-fibrotic effects of KP-13, we measured the transcript levels of *Col1*, *Mmp9*, and alpha-smooth muscle actin (*Acta2*) in human ventricular cardiac fibroblasts (HVCFs) exposed to TGF-β in the presence or absence of KP-13 (Figure S4). TGF-β exposure was accompanied by a statistically non-significant, slight elevation of the *Col1* transcript levels (*p* = 0.159), whereas no significant difference was detected between the KP-13-treated and control groups in HVCFs (Figure S4). *Mmp9* and *Acta2* expressions showed no significant differences between the groups in HVCFs (Figure S4)*.*

### Effects of KP-13 on pERK/ERK ratios in CKD

**Fig. 7:**
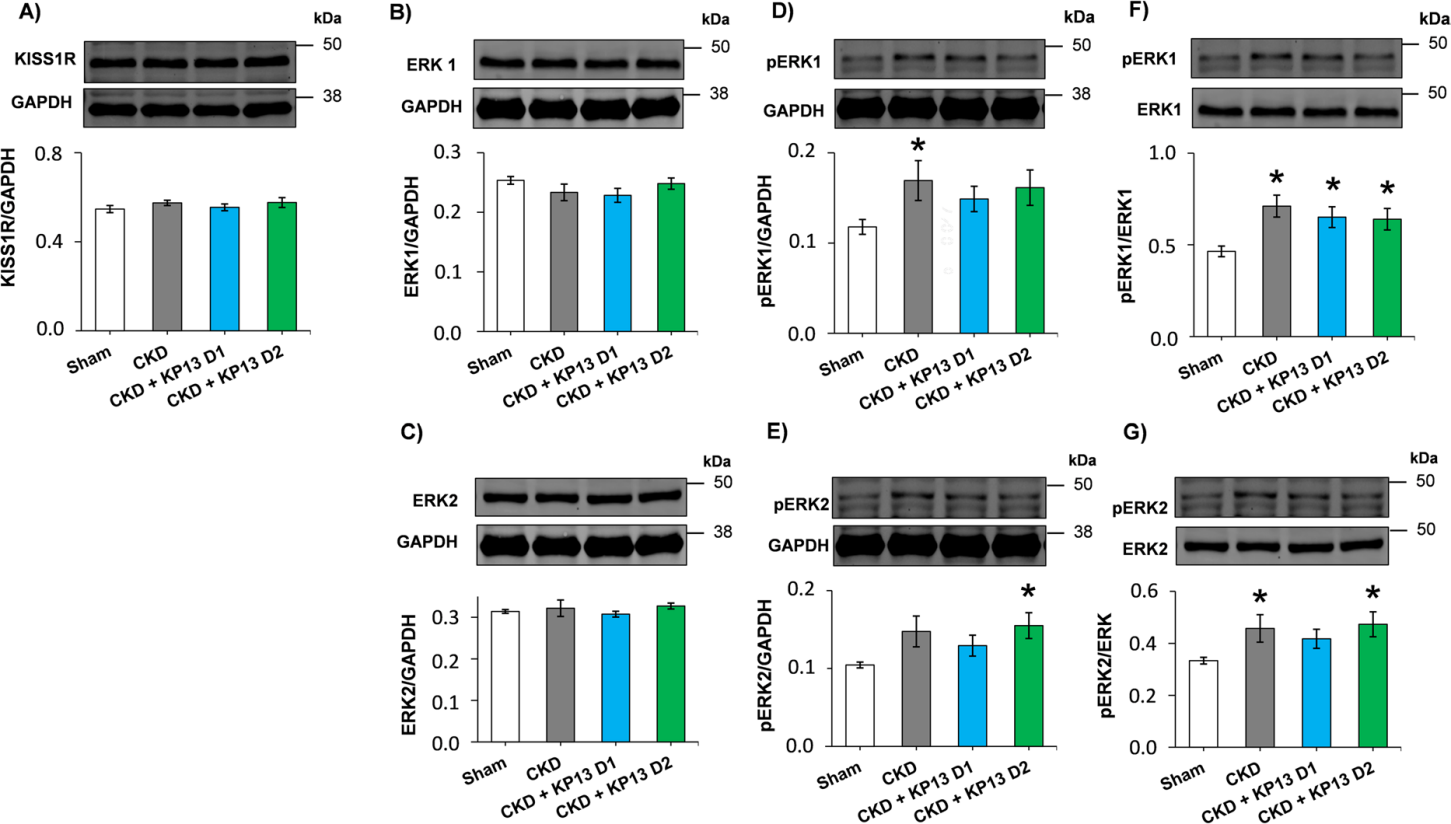
The effects of the KISS1R agonist kisspeptin-13 on the protein levels of KISS1R and ERK1/2 at week 13. Left ventricular protein levels and cropped representative Western blot images of **A** kisspeptin receptor-1 (KISS1R, 40–140 kDa), **B** total ERK1 (44 kDa), **C** total ERK 2 (42 kDa), **D** phospho-ERK1 (pERK1, 44 kDa), **E** phospho-ERK2 (pERK2, 42 kDa), **F** pERK1/ERK1 ratio, and **G** pERK2/ERK2 ratio. Values are presented as mean ± SEM., *p < 0.05 vs. sham (*n* = 7, one-way ANOVA, Holm-Sidak post hoc test). Sham, sham-operated group; CKD, chronic kidney disease group; CKD + KP-13 D1, chronic kidney disease group treated with the lower dose (13 µg/day, dose 1) of the KISS1R agonist kisspeptin-13; CKD + KP-13 D2, chronic kidney disease group treated with the higher dose (26 µg/day, dose 2) of the KISS1R agonist kisspeptin-13. Images were captured with the Odyssey CLx machine and exported with Image Studio 5.2.5 software

ERK1/2 could be activated via the KISS1R [50]. In our present study, KISS1R protein was expressed in the LV tissue, and its levels were not significantly different between the groups (Fig. 7A, Figure S5). It is well known that the activation of the ERK1/2 signaling pathway can induce cardiac hypertrophy and fibrosis [51, 52]. There were no significant differences between the total ERK1 and ERK2 levels between the groups (Fig. 7B and C, Figure S6). However, pERK1 level and pERKs/ERKs ratios were significantly increased (*p < 0.05), and pERK2 level tended to increase (*p* = 0.053) in the CKD-only group compared to the sham-operated group (Fig. 7D, E, F, and G, Figure S7).

In the lower dose of the KP-13-treated CKD group, ERK1 showed a trend to decrease (*p* = 0.085), the pERK1/ERK ratio remained significantly increased, the pERK1 level and the pERK2/ERK2 ratio tended to increase (*p* = 0.081 and *p* = 0.051 respectively), and pERK2 level was not different compared to the sham-operated group (Fig. 7B, D, E, F, and G, Figures S6 and S7). In the higher dose of the KP-13-treated CKD group, both the pERK/ERK ratio and pERK2 level showed a significant increase (*p < 0.05), and the pERK1 level tended to increase (*p* = 0.063) compared to the sham-operated group (Fig. 7D, E, F, and G, Figures S6 and S7).

### KP-13 increases Bax/Bcl2 ratio in CKD

**Fig. 8:**
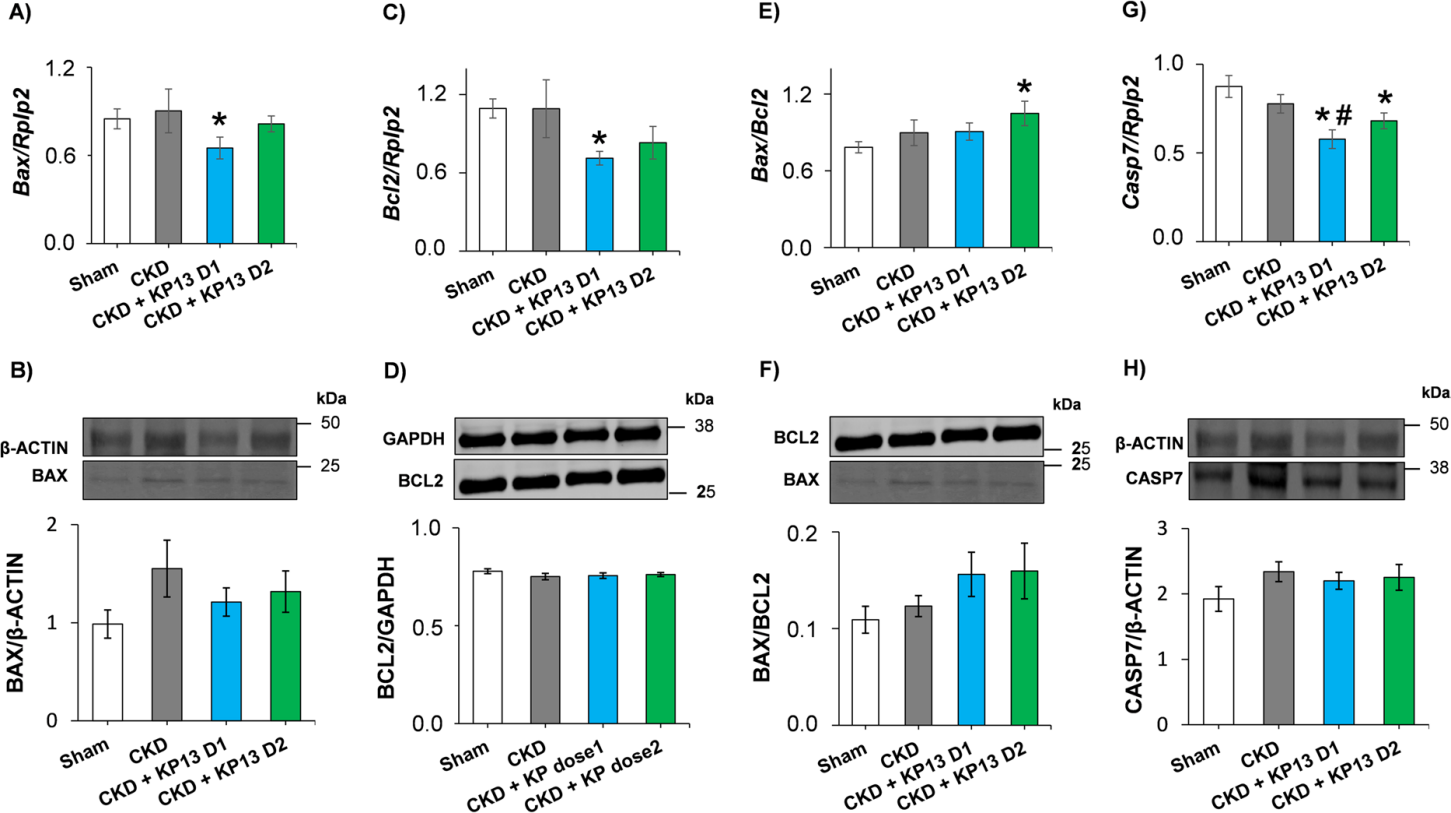
The effects of the KISS1R agonist kisspeptin-13 on apoptosis-associated gene expressions and protein levels in the left ventricles at week 13. Left ventricular relative gene expressions, protein levels, and cropped representative images of **A** BCL2-associated X apoptosis regulator (*Bax*), **B** BAX (20 kDa), **C** B-cell CLL/lymphoma 2 apoptosis regulator (*Bcl2*), **D** BCL2 (26 kDa), **E** *Bax/Bcl2* ratio, **F** BAX/BCL2 ratio, **G** caspase7 (*Casp7*), and **H** CASP7 (35 kDa). Values are presented as mean ± SEM, *p < 0.05 vs. sham, # *p* < 0.05 vs. CKD vehicle group (*n* = 7–8 for RT-qPCR and *n* = 7 for Western blot measurements, one-way ANOVA, Holm-Sidak *post* hoc test). Sham, sham-operated group; CKD, chronic kidney disease group; CKD + KP-13 D1, chronic kidney disease group treated with the lower dose (13 µg/day, dose 1) of the KISS1R agonist kisspeptin-13; CKD + KP-13 D2, chronic kidney disease group treated with the higher dose (26 µg/day, dose 2) of the KISS1R agonist kisspeptin-13. Gene expression is normalized to the ribosomal protein lateral stalk subunit P2 (*Rplp2*) gene expression. Western blot images were captured with the Odyssey CLx machine and exported with Image Studio 5.2.5 software

On the route to heart failure, pathological cardiac hypertrophy progresses to the decompensation phase typically associated with cardiomyocyte apoptosis [53]. In our present study, there were no significant differences in the LV expressions of selected apoptosis-associated markers (*Bax, Bcl2*, *Bax/Bcl2* ratio, and *Casp7*) between the CKD-only and sham-operated groups at the transcript or protein levels at this phase of uremic cardiomyopathy at week 13 (Fig. 8A, B, C, D, E, F, G, and H, Figures S8–S10). However, it should be mentioned that BAX and CASP7 protein levels tended to increase (*p* = 0.126 and *p* = 0.116, respectively) in the CKD-only group compared to the sham-operated group (Fig. 8B and H, Figures S8 and S10).

In the lower dose of the KP-13-treated CKD group, the *Bax, Bcl2, and Casp7* expressions were significantly decreased compared to the sham-operated group (*p < 0.05) (Fig. 8A, C, and G). Moreover, *Casp7* was significantly repressed in the lower dose of the KP-13-treated CKD group compared to the CKD-only group (^#^*p* < 0.05) (Fig. 8G). Notably, in the lower dose of the KP-13-treated CKD group, the Bax/Bcl2 ratio showed an increasing tendency at the mRNA and protein levels (*p* = 0.079 and *p* = 0.121, respectively) compared to the sham-operated group (Fig. 8E and F, Figures S8 and S9). In the higher dose of the KP-13-treated CKD group, the expression of *Casp7* was significantly decreased (*p < 0.05), and the expression of *Bcl2* was tendentiously decreased (*p* = 0.077) compared to the sham-operated group (*p < 0.05) (Fig. 8C and G). There were no significant differences in the protein levels of BCL2 and CASP7 between the groups (Fig. 8D and H, Figures S9 and S10). However, the *Bax/Bcl2* ratio was significantly increased at the mRNA level and tended to increase at the protein level (*p* = 0.146) in the higher dose of KP-13-treated CKD group compared to the sham-operated group (Figs. 8E and F, Figures S8 and S9).

## Discussion

The present study unveils a novel perspective by demonstrating, for the first time, the impact of the KISS1R agonist KP-13 on systemic blood pressure, crucial renal parameters, and cardiac function within a 5/6 nephrectomy-induced rat model of CKD. We show that KP-13 increased systemic blood pressure and worsened the serum urea levels, glomerular hypertrophy, and tubular dilation in this preclinical model of CKD. Remarkably, treatment with a higher dose of KP-13 correlated with reduced posterior and anterior wall thickness, as well as diminished cardiomyocyte cross-sectional areas in CKD. These structural changes were concomitant with deteriorating echocardiographic indicators of diastolic dysfunction, alongside an increased expression of inflammatory (*Il6, Tnf*), fibrosis (*Col1*), and apoptosis-related markers (*Bax/Bcl2*). Intriguingly, the administration of KP-13 yielded contrasting results when evaluated against circulatory uremic toxin levels, tissue ACE activity, renal inflammatory and fibrosis markers, and renal and LV interstitial fibrosis in the context of CKD. This intricate interplay of effects uncovers previously unexplored facets of the KISS1R agonist’s impact on CKD-related parameters, furthering our understanding of the mechanisms contributing to uremic cardiomyopathy.

Our study brings to light a series of interwoven findings, each contributing to the intricate landscape of CKD and uremic cardiomyopathy. Initially, our investigation established characteristic CKD laboratory markers, including elevated serum urea and creatinine levels, as well as a decline in creatinine clearance by week 5 in the 5/6 nephrectomy-induced CKD model. These alterations, indicative of early-stage CKD, are typically detectable through routine clinical laboratory screening before the emergence of uremic cardiomyopathy, marked by LVH and associated fibrosis. It is noteworthy that our previous research demonstrated comparable LV morphology and function in both the sham-operated and CKD-only groups at the 2-week mark post-5/6 nephrectomy [34]. Thus, to examine the effects of KP-13 during the initial asymptomatic CKD phase, devoid of LVH, we commenced KP-13 administration in the third week after CKD induction.

In healthy rats, the primary function of kisspeptin is thought to be the regulation of the hypothalamic-pituitary–gonadal axis; therefore, the effects of the different forms of kisspeptin were mainly investigated in connection with reproductive function [54]. In the case of chronic administration of high-dose kisspeptins, tachyphylaxis on the hypothalamic-pituitary–gonadal axis developed [55]. Moreover, KP-KISS1R signaling has an effect on the expression of metabolic genes involved in appetite control [56], as well as it seems to have a crucial role in the control of insulin secretion [57]. Indeed, at the endpoint of our study, the KP-13-treated CKD groups showed reduced weight gain compared to the sham-operated group. This result warns that a continuous high dose of KP-13 treatment could more likely induce metabolic complications in CKD.

At the experimental endpoint, our present findings align CKD severity with human G2 or G3a stages, reflecting a moderate reduction in kidney function [58, 59]. This consistency with our prior research reinforces the reliability of our CKD model [28, 34, 36]. While the involvement of KPs-KISS1R signaling in CKD pathophysiology remains unclear, significant insights emerge from previous studies. Shoji et al. demonstrated elevated KP concentrations in the remnant kidneys of rats 8 weeks post 5/6 nephrectomy, implying the possible role of KPs in CKD development [14]. In contrast, renal KISS1R protein levels decreased due to the persistently elevated kisspeptin levels [14]. Our results align with this narrative, revealing that exogenous administration of the KISS1R agonist KP-13 led to augmented glomeruli and tubular dimensions, aligning with Yi et al.’s findings on the link between KISS1R-mediated signaling and glomerular development [60]. Moreover, the lower KP-13 dose further elevated serum urea levels and exhibited a tendency to decrease creatinine clearance in CKD, mirroring Luedde et al.’s observation of a strong correlation between circulating kisspeptin levels and decreased renal function parameters in critically ill patients [61].

Severe hypertension is not a prominent feature in the 5/6 nephrectomy-induced rat CKD model [62–64], and our model similarly exhibited only a slight rise in systolic, diastolic, and mean arterial blood pressure. Therefore, it seems plausible that LVH, fibrosis, and diastolic dysfunction development might not be primarily attributable to elevated blood pressure in the CKD-only group. However, both doses of KP-13 significantly increased systemic blood pressure in CKD in the present study. This aligns with Mead et al.’s findings that KPs acted as vasoconstrictors, akin in potency to angiotensin-II in the human aorta and coronary arteries [31]. Consequently, the vasoconstrictor role of KP-13 emerges as a potential factor in worsening renal morphology within our CKD model. However, the investigation of the potential vasoconstrictor effects of KP-13 on renal arteries would also be desirable in future studies.

The RAAS is a well-known player in the development of uremic cardiomyopathy and CKD [8]. Therefore, the renal *Agtr2* expression and ACE activity was assessed. In consistence with previous studies [64, 65], ACE activity was tendentiously decreased in the CKD-only group in our present study, probably, due to the renal endothelial cell damage [64]. Furthermore, in the lower dose of the KP-13-treated group, the ACE activity showed an increasing tendency compared to the CKD-only group, suggesting that KP-13 might have an effect on the RAAS.

The CKD-only group demonstrated echocardiography, histology, and autopsy outcomes in alignment with our prior findings of LVH development, diastolic dysfunction, preserved EF, and LV interstitial fibrosis in the 5/6 nephrectomy-induced CKD model [28, 34, 36, 37, 39]. In contrast, KP-13 administration’s effects proved nuanced. The CKD group treated with the lower KP-13 dose demonstrated similarities to the CKD-only group with respect to LVH severity, diastolic dysfunction, and preserved systolic function. Interestingly, LV collagen content was lower in response to the lower KP-13 dose in CKD, mirroring previous findings on KP-13’s anti-inflammatory and anti-fibrotic impact [29, 30]. The higher KP-13 dose, in contrast, exhibited diminished hypertrophic signs, associated markers, and more pronounced diastolic dysfunction in CKD. This group’s elevated expression of inflammatory and apoptotic markers potentially explains this lesser degree of LVH, indicating a transition toward eccentric LV remodeling.

While uremic toxins are implicated in LVH and fibrosis among CKD patients [47, 48], our findings do not substantiate a direct relationship among KP-13, uremic toxins, and LV or kidney morphology, as serum uremic toxin levels remained consistent across CKD groups.

ANP and BNP are autocrine/paracrine inhibitors of cardiac hypertrophy and collagen synthesis [66, 67]. The KP-13-induced dynamics of *Nppa* and *Nppb*, encoding ANP and BNP, respectively, also provided insights. The lower KP-13 dose correlated with increased *Nppa* expression in CKD, in contrast to the higher dose, which demonstrated reduced *Nppa* and *Nppb* expressions in CKD. This intricate regulation reflects the complex interplay between these hormones and mediators, their counter-regulatory roles, and the progression of hypertrophy.

We have recently shown that the KISS1R antagonist P234 led to reduced anterior and posterior wall thicknesses and more severe interstitial fibrosis in the identical CKD model via a mechanism involving TGF-β-mediated signaling pathways [28]. In contrast, in our present study, KP-13 (100 ng/mL) failed to alter the expression of key fibrosis genes in human ventricular cardiac fibroblasts. Accordingly, in our present study, the TGF-β-mediated pathways, central to renal and cardiac fibrosis [68], do not seem directly influenced by KP-13 administration, considering similar expression levels in all CKD groups. There was no significant difference in the severity of renal fibrosis among the CKD groups, and only the lower dose of KP-13 reduced the LV fibrosis in CKD. Similarly, the ERK1/2 signaling pathway, associated with cardiac hypertrophy and fibrosis [51, 52], remains active in CKD independently of KP-13. These findings underscore the multifaceted nature of pathways contributing to CKD and uremic cardiomyopathy.

In this study, we aimed to investigate the potential effects of KP-13 on renal and LV morphology and function using a rat model of CKD. However, it is important to acknowledge that there are significant differences between experimental and clinical CKD and uremic cardiomyopathy. Our use of juvenile inbred male rats without comorbidities such as atherosclerosis, obesity, and diabetes mellitus may not fully capture the complexity of the clinical scenario. Moreover, the 5/6-nephrectomized rat model used in this study may exhibit less pronounced hypertension and earlier heart failure development compared to the clinical setting. Additionally, we only used male rats in the study, despite the possibility of female sex hormones influencing the progression and severity of CKD and uremic cardiomyopathy.

The kisspeptin-KISS1R system has garnered significant interest in the context of aging, with emerging evidence suggesting its involvement in age-related physiological changes [16, 18, 69–77]. Kisspeptins synthesized in the hypothalamus regulate the endocrine functions of the pituitary. Notably, the diminishing kisspeptin levels with advancing age could potentially contribute to a mechanism through which the central nervous system orchestrates systemic aging processes, aligning with the premise of the neuroendocrine theory of aging [15]. Research has revealed that the expression of both kisspeptins and KISS1R is dysregulated with advancing age in various tissues, including the brain and peripheral organs [16, 74, 78]. This age-related decline may contribute to alterations in reproductive function and hormone regulation [19, 70, 79, 80]. Importantly, the kisspeptin-KISS1R system has been implicated in the regulation of the hypothalamic-pituitary–gonadal axis, which plays a crucial role in the aging process [19, 70, 79, 80]. Research has also explored the potential of kisspeptins to modulate inflammatory processes, which hold implications for diverse age-related conditions. To mirror the clinical context more authentically, future studies could incorporate animal models of both sexes, encompassing relevant comorbidities and advanced aging. While our primary focus lay on the effects of the KISS1R agonist KP-13, delving into the impacts of KISS1R antagonists and other agonists would be of considerable value, enhancing our comprehension of their potential implications for uremic cardiomyopathy and CKD within the aging framework. Such holistic research will yield a more nuanced grasp of the kisspeptin-KISS1R system’s role in age-associated conditions. The precise mechanisms through which this system influences cellular and molecular aging processes remain partially obscured, warranting further exploration. A comprehensive understanding of this system’s significance in the biology of aging and its potential ramifications for age-related health outcomes and interventions remains a crucial avenue of investigation. For instance, the interplay of kisspeptin-KISS1R system and age-related alterations in cellular mechanisms, such as induction of cellular senescence [81], compromised autophagy [77, 82], and stress resilience [76, 83–85], impaired proteastasis, impaired mitochondrial function [77] and heightened oxidative stress [86], exacerbating the development of CKD and uremic cardiomyopathy and their progression among aging individuals warrants additional investigations. Unraveling the influence of the kisspeptin-KISS1R axis on these aging-related cellular mechanisms [77] and their contributions to CKD pathogenesis may hold importance for devising effective preventive and therapeutic strategies to address the mounting burden of CKD in the aging population. Moreover, kisspeptin is believed to play a role in the regulation of metabolism by exerting anti-obesitogenic effects, and its levels are inversely correlated with insulin resistance [17]. These metabolic pathways also hold significance in the regulation of cellular aging processes. Consequently, exploring the systemic metabolic impacts of pharmacological interventions that modulate the activity of the kisspeptin-KISS1R axis within the realm of aging biology should be a subject of investigation.

In summary, the activation of the kisspeptin-KISS1R system holds the potential to significantly contribute to the pathogenesis of CKD and uremic cardiomyopathy. Its influence on blood pressure regulation and the exacerbation of inflammatory and apoptotic pathways seems pivotal in this context. These insights underscore the promise of investigating the kisspeptin-KISS1R axis in future studies as a means to develop preventive strategies aimed at mitigating the initiation and advancement of CKD and uremic cardiomyopathy.

### Supplementary Information

Below is the link to the electronic supplementary material.

Supplementary file1 (DOCX 4604 KB)

## Data Availability

The datasets used and/or analyzed during the current study are available from the corresponding authors upon reasonable request.
