# Supplementary material for: Role of the kisspeptin-KISS1R axis in the pathogenesis of chronic kidney disease and uremic cardiomyopathy
Source: GeroScience. 2023 Nov 21;46(2):2463–88. doi: 10.1007/s11357-023-01017-8 (PMC10828495; doi:10.1007/s11357-023-01017-8)
Supplement: Supplementary file 1 — Supplementary file1 (DOCX 4604 KB) [file 11357_2023_1017_MOESM1_ESM.docx]

**Supplementary Material**

**Role of the Kisspeptin-KISS1R axis in the pathogenesis of chronic kidney disease and uremic cardiomyopathy**

Hoa Dinh^1,2^ [dinhhoaqa@gmail.com](mailto:dinhhoaqa@gmail.com), ORCID: 0000-0001-5812-715X

Zsuzsanna Z.A. Kovács^1^ [zsuzska.k93@gmail.com](mailto:zsuzska.k93@gmail.com), ORCID: 0000-0002-4197-4579

Merse Kis^1,3^ [kissmerse@gmail.com](mailto:kissmerse@gmail.com), ORCID: 0009-0006-3125-4670

Klaudia Kupecz^1,3^ [kupeczklau@gmail.com](mailto:kupeczklau@gmail.com), ORCID: 0009-0004-8128-3280

Anita Sejben^4^ [sejben.anita@med.u-szeged.hu](mailto:sejben.anita@med.u-szeged.hu), ORCID: 0000-0002-9434-2989

Gergő Szűcs^1^ [szucs.gergo@med.u-szeged.hu](mailto:szucs.gergo@med.u-szeged.hu), ORCID: 0000-0003-1874-2718

Fanni Márványkövi^1^ [marvanykovifanni@gmail.com](mailto:marvanykovifanni@gmail.com), ORCID: 0000-0002-5114-1319

Andrea Siska^5^ [siska.andrea@med.u-szeged.hu](mailto:siska.andrea@med.u-szeged.hu), ORCID: 0000-0002-2252-7095

Marah Freiwan^1^ [marah.mf.94@gmail.com](mailto:marah.mf.94@gmail.com), ORCID: 0000-0002-2482-0367

Szonja Polett Pósa^1^ [posszonja@gmail.com](mailto:posszonja@gmail.com), ORCID: 0000-0002-7535-9689

Zsolt Galla^6^ [gallazs87@gmail.com](mailto:gallazs87@gmail.com), ORCID: 0000-0002-9166-1212

Katalin Eszter Ibos^3^ [ibos.katalin.eszter@med.u-szeged.hu](mailto:ibos.katalin.eszter@med.u-szeged.hu), ORCID: 0000-0001-5243-9945

Éva Bodnár^3^ [eva.dobo4@gmail.com](mailto:eva.dobo4@gmail.com), ORCID: 0000-0002-7938-8719

Gülsüm Yilmaz Lauber^7^ [guelsuem.yilmazlauber@meduniwien.ac.at](mailto:guelsuem.yilmazlauber@meduniwien.ac.at)

Ana Isabel Antunes Goncalves^7^ [ana.antunesgoncalves@meduniwien.ac.at](mailto:ana.antunesgoncalves@meduniwien.ac.at)

Eylem Acar^7^ [eylem.acar@meduniwien.ac.at](mailto:eylem.acar@meduniwien.ac.at), ORCID: 0000-0002-0599-6893

András Kriston^8,9,10^ [kriston.andras@single-cell-technologies.com](mailto:kriston.andras@single-cell-technologies.com), ORCID: 0000-0001-8500-4315

Ferenc Kovács^8,9,10^ [kovacs.ferenc@single-cell-technologies.com](mailto:kovacs.ferenc@single-cell-technologies.com), ORCID: 0000-0003-4512-4448

Péter Horváth^8,9,10^ [horvath.peter@brc.hu](mailto:horvath.peter@brc.hu), ORCID: 0000-0002-7355-2936

Zsolt Bozsó^11^ [bozso.zsolt@med.u-szeged.hu](mailto:bozso.zsolt@med.u-szeged.hu), ORCID: 0000-0002-5713-3096

Gábor Tóth^11^ [toth.gabor@med.u-szeged.hu](mailto:toth.gabor@med.u-szeged.hu)

Imre Földesi^4^ [foldesi.imre@med.u-szeged.hu](mailto:foldesi.imre@med.u-szeged.hu), ORCID: 0000-0002-3329-8136

Péter Monostori^5^ [monostoripeter@gmail.com](mailto:monostoripeter@gmail.com), ORCID: 0000-0003-3591-6054

Gábor Cserni^3^ [cserni.gabor@med.u-szeged.hu](mailto:cserni.gabor@med.u-szeged.hu), ORCID: 0000-0003-1344-7744

Bruno K. Podesser^7^ [bruno.podesser@meduniwien.ac.at](mailto:bruno.podesser@meduniwien.ac.at), ORCID: 0000-0002-4641-7202

Andrea Lehoczki^12^ [ceglediandi@freemail.hu](mailto:ceglediandi@freemail.hu), ORCID: 0000-0002-4285-7518

Peter Pokreisz^7^ [peter.pokreisz@meduniwien.ac.at](mailto:peter.pokreisz@meduniwien.ac.at), ORCID: 0000-0003-2810-9000

Attila Kiss^7^ [attila.kiss@meduniwien.ac.at](mailto:attila.kiss@meduniwien.ac.at), ORCID: 0000-0003-4652-1998

László Dux^1*^ [dux.laszlo@med.u-szeged.hu](mailto:dux.laszlo@med.u-szeged.hu), ORCID: 0000-0002-1270-1678

Krisztina Csabafi^3#^ [csabafi.krisztina@med.u-szeged.hu](mailto:csabafi.krisztina@med.u-szeged.hu), ORCID: 0000-0002-2008-7604

Márta Sárközy^1,3*#^ [martasarkozy@gmail.com](mailto:martasarkozy@gmail.com), ORCID: 0000-0002-5929-2146

^1^Department of Biochemistry and Interdisciplinary Centre of Excellence, Albert Szent-Györgyi Medical School, University of Szeged, H-6720 Szeged, Hungary

^2^Department of Biochemistry, Bach Mai Hospital, Hanoi, 100000, Vietnam

^3^Department of Pathophysiology, Albert Szent-Györgyi Medical School, University of Szeged, Szeged, H-6720, Hungary

^4^Department of Pathology, Albert Szent-Györgyi Medical School, University of Szeged, Szeged, H-6720, Hungary

^5^Department of Laboratory Medicine, Albert Szent-Györgyi Medical School, University of Szeged, H-6720 Szeged, Hungary

^6^Metabolic and Newborn Screening Laboratory, Department of Pediatrics, Albert Szent-Györgyi Medical School, University of Szeged, H-6720 Szeged, Hungary

^7^Ludwig Boltzmann Institute for Cardiovascular Research at Center for Biomedical Research and Translational Surgery, Medical University of Vienna, Vienna, A-1090, Austria

^8^Synthetic and Systems Biology Unit, Biological Research Centre, Eötvös Loránd Research Network, H-6726 Szeged, Hungary

^9^Single-Cell Technologies Ltd, Szeged, H-6726, Hungary,

^10^Institute for Molecular Medicine Finland (FIMM), University of Helsinki, 00014, Helsinki, Finland

^11^Department of Medical Chemistry, Albert Szent-Györgyi Medical School, University of Szeged, H-6720 Szeged, Hungary

^12^Departments of Hematology and Stem Cell Transplantation, South Pest Central Hospital, National Institute of Hematology and Infectious Diseases, Saint Ladislaus Campus, Budapest, Hungary

*Correspondence: [martasarkozy@gmail.com](mailto:martasarkozy@gmail.com) and [dux.laszlo@med.u-szeged.hu](mailto:dux.laszlo@med.u-szeged.hu)

^#^These authors contributed equally to the work

**(A)**


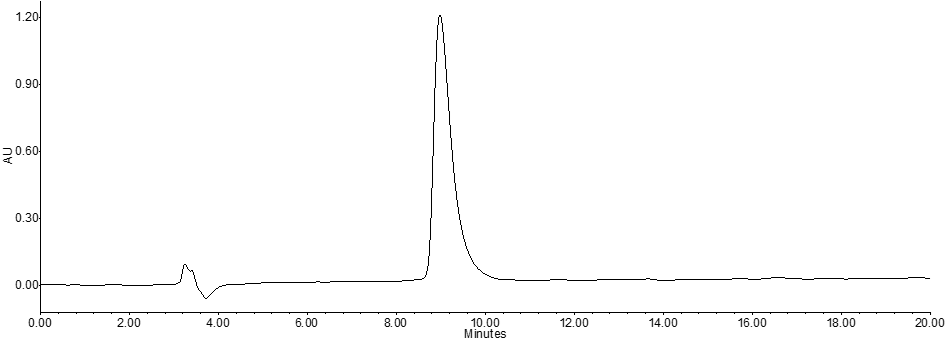


**(B)**


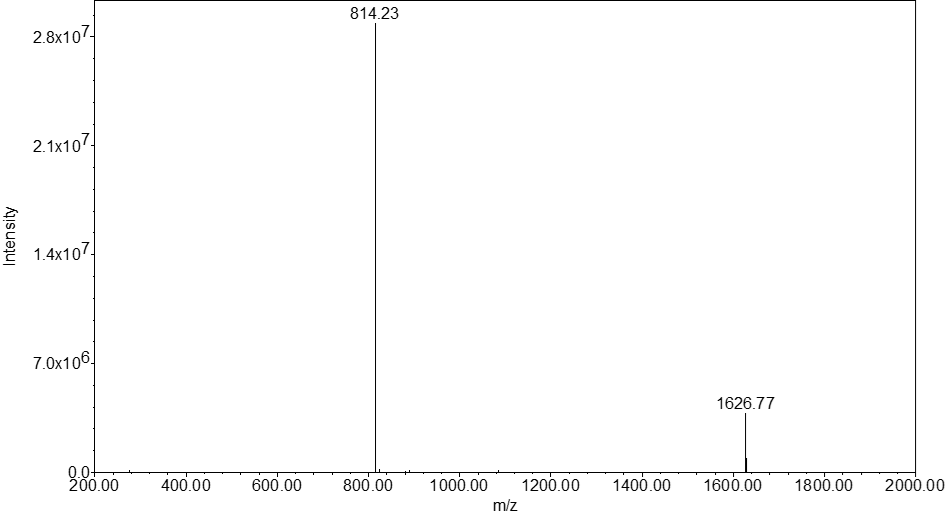


**Figure S1** (A) HPLC trace of purified Kisspeptin-13, gradient: 40-60% eluent B in eluent A over 20 min, 1 mL/min, 210 nm, (B) mass spectrum of Kisspeptin-13.

(A)

(B)

**Figure S2** The effects of the KISS1R agonist kisspeptin-13 on (A) renal arteriola hyalinosis and (B) chronic pyelonephritis at week 13. Sham: sham-operated group, CKD: chronic kidney disease group, CKD + KP-13 D1: chronic kidney disease group treated with the lower dose (13 μg/day, Dose 1) of the KISS1R agonist kisspeptin-13, CKD + KP-13 D2: chronic kidney disease group treated with the higher dose (26 μg/day, Dose 2) of the KISS1R agonist kisspeptin-13. Fisher's test was carried out for renal histology scoring using R software (version 4.2.3 for Windows) to compare outcome measures between treatment groups (n=6-8). Fisher's test revealed a statistically significant association between KP-13 treatment and arteriola hyalinosis (p=0.0001744), as well as chronic pyelonephritis (P=0.00006935).

**Transcription profiling by RT-qPCR from kidney samples**

Total RNA was extracted from kidney samples using the RNeasy Mini Kit (Qiagen, Hilden, Germany), quantified by Tecan reader using the NanoQuant plate, and 2,5 μg of total RNA was reverse transcribed using the RevertedAid First Strand cDNA Synthesis Kit (ThermoScientific, USA). Samples were analyzed in technical duplicates using a 20 μL reaction volume with a 1:5 dilution of cDNA in master mix solution. The initial denaturation step of 3 min at 95 °C was followed by 40 cycles of 15 s at 95 °C, 30 s at 60 °C, and 40 s at 72 °C, using a CFX-Opus96 thermocycler with the accompanying CFX Manager software (Bio-Rad Laboratories Inc., USA) for relative quantification using the Cq values. According to the MIQE guidelines, not Ct but the term quantification cycle (Cq) was used (Bustin et al. 2009). All Cq values calculated by the cycler software, differences between the two technical replicates of all samples are given in Figure S2.

Specific rat primers (*Kiss1:* kisspeptin 1; *Agtr2*: angiotensin type 2 receptor) (Microsynth GmbH, Austria) and PerfeCTa SYBR Green FastMix (QuantaBio, USA) were used according to the manufacturer’s instructions to produce the master mix solution.

| **Primer** | **Forward Sequence 5’-3’** | **Reverse Sequence 5’-3’** |
| --- | --- | --- |
| *Kiss1* | TCACCAATTTCTACATCGCTAAC | CACGCAGCGGGAACACAG |
| *Agtr2* | CCATCCAGGTCAGAGCATGC | AGGAATCCGTGGCAAGCATC |

**Reference**

Bustin, Stephen A.; Benes, Vladimir; Garson, Jeremy A.; Hellemans, Jan; Huggett, Jim; Kubista, Mikael et al. (2009): The MIQE guidelines: minimum information for publication of quantitative real-time PCR experiments. In *Clinical chemistry* 55 (4), pp. 611–622. DOI: 10.1373/clinchem.2008.112797.

**
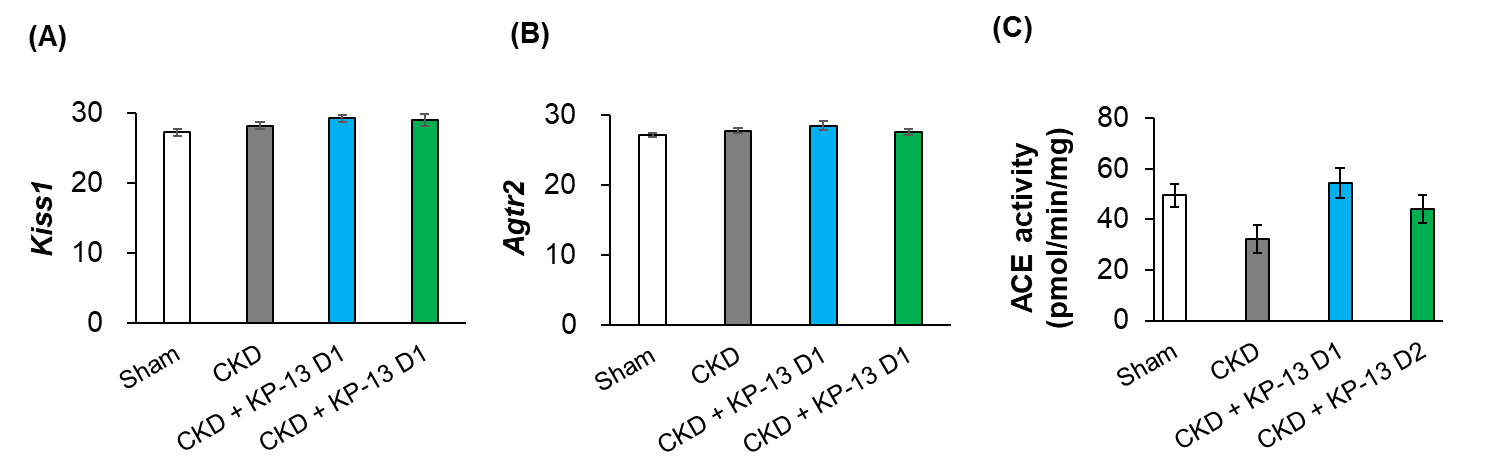
**

**Figure S3** The effects of the KISS1R agonist kisspeptin-13 on (A) renal ACE activity, and expressions of (B) kisspeptin (*Kiss1*) and (C) angiotensin II receptor type 2 (*Agtr2*). Sham: sham-operated group, CKD: chronic kidney disease group, CKD + KP-13 D1: chronic kidney disease group treated with the lower dose (13 μg/day, Dose 1) of the KISS1R agonist kisspeptin-13, CKD + KP-13 D2: chronic kidney disease group treated with the higher dose (26 μg/day, Dose 2) of the KISS1R agonist kisspeptin-13. (n=6, One-Way ANOVA, Holm-Sidak *post hoc* test).


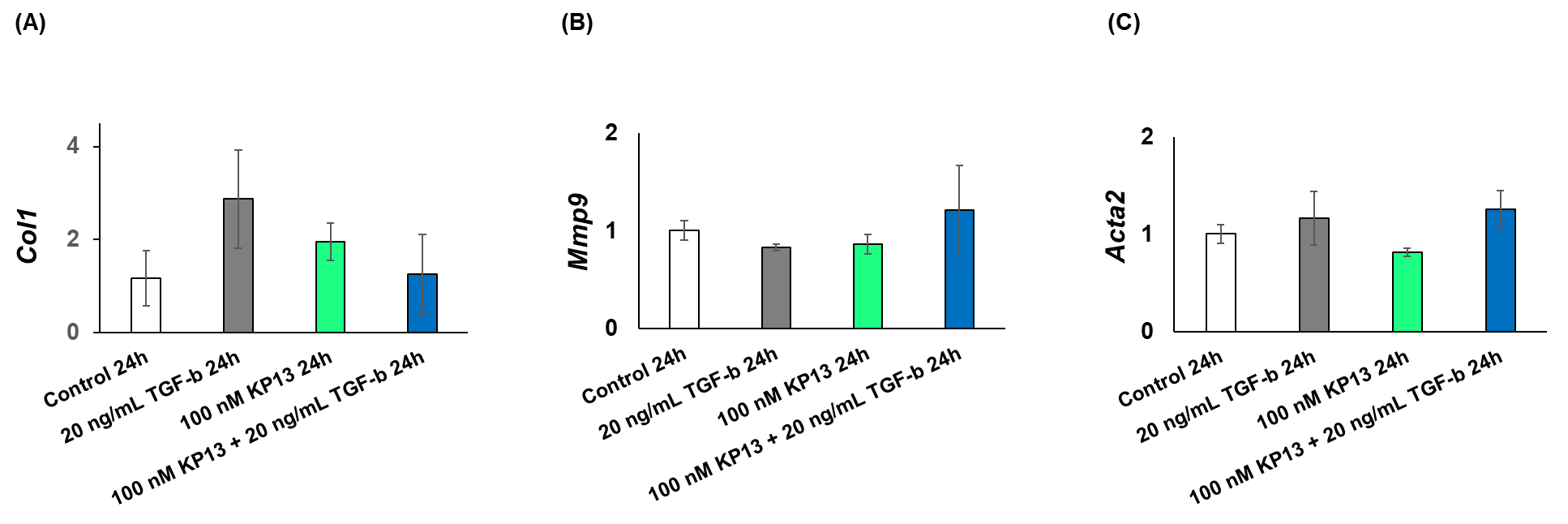


**Figure S4** The effects of KP-13 with or without TGF-β on the expression of (A) collagen-1 (*Col1*)*,* (B) matrix metalloprotease-9 (*Mmp9*)*,* and (C) α-smooth muscle actin (*Acta2*) in human ventricular cardiac fibroblasts HVCFs. Values are presented as mean ± S.E.M., * p < 0.05 vs. sham (n = 3, One-Way ANOVA, Bonferroni *post hoc* test).


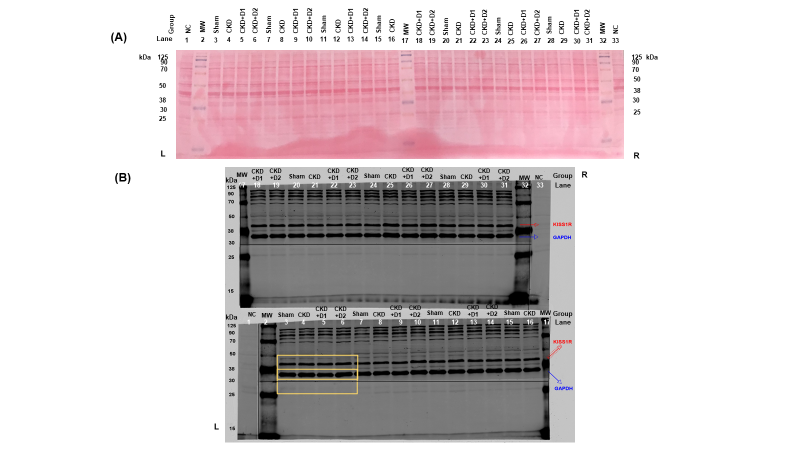


**Figure S5** Unmodified Ponceau-stained membranes and Western blot images of KISS1R and GAPDH. (A) Photo of the uncropped and unmodified Ponceau-stained membrane used later for the detection of KISS1R and GAPDH. The efficacy of the transfer of proteins onto a nitrocellulose membrane was checked using Ponceau staining. Images were captured by the camera of an Apple iPhone 7 plus. (B) Unmodified Western blot images of KISS1R and GAPDH. Images were captured with the Odyssey CLx machine and exported with Image Studio 5.2.5 software. Bands in yellow boxes are shown in Fig. 6 as representative blots. Sham: sham-operated group, CKD: chronic kidney disease group, CKD+D1: chronic kidney disease group treated with the lower dose (13 μg/day, dose 1) of KISS1R agonist KP-13, CKD+D2: chronic kidney disease group treated with the higher dose (26 μg/day, dose 2) of KISS1R agonist KP-13. MW: molecular weight marker, NC: negative control, L: left, R: right.

**
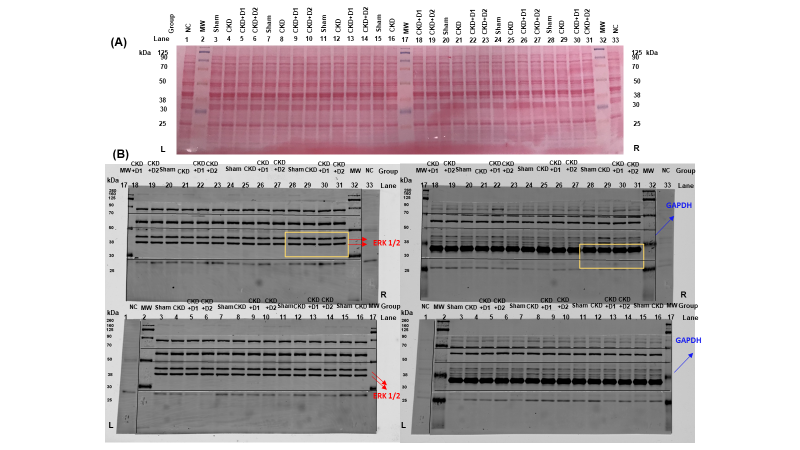
**

**Figure S6** Unmodified Western blot images of ERK 1/2 and GAPDH. (A) Photo of the uncropped and unmodified Ponceau-stained membrane used later for the detection of ERK 1/2 and GAPDH. The efficacy of the transfer of proteins onto a nitrocellulose membrane was checked using Ponceau staining. Images were captured by the camera of an Apple iPhone 7 plus. (B) Unmodified Western blot images of ERK 1/2 and GAPDH. Images were captured with the Odyssey CLx machine and exported with Image Studio 5.2.5 software. Bands in yellow boxes are shown in Fig. 6 as representative blots. Sham: sham-operated group, CKD: chronic kidney disease group, CKD+D1: chronic kidney disease group treated with the lower dose (13 μg/day, dose 1) of KISS1R agonist KP-13, CKD+D2: chronic kidney disease group treated with the higher dose (26 μg/day, dose 2) of KISS1R agonist KP-13. MW: molecular weight marker, NC: negative control, L: left, R: right.


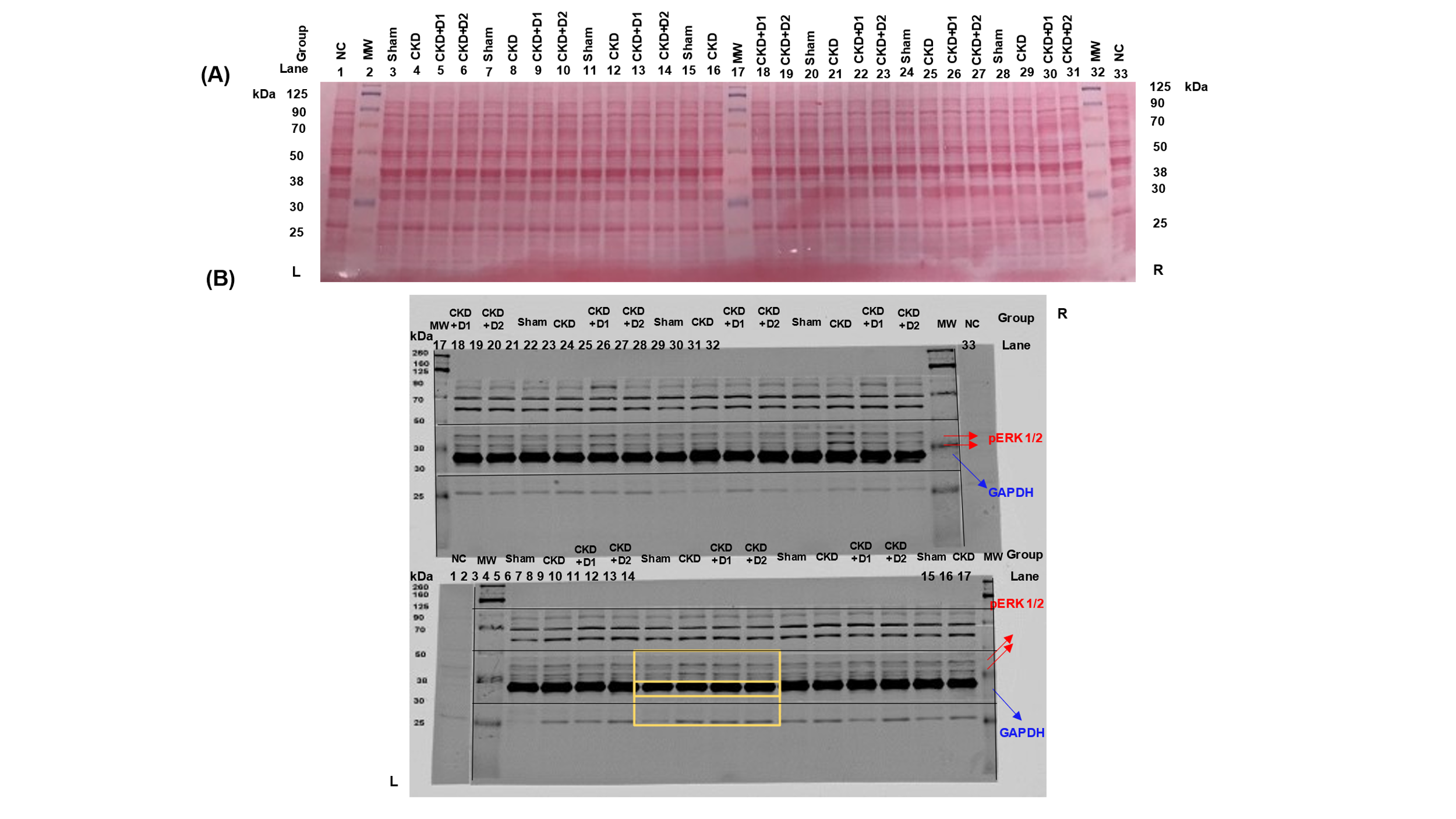


**Figure S7** Unmodified Western blot images of phospho-ERK 1/2 (pERK 1/2) and GAPDH**.** (A) Photo of the uncropped and unmodified Ponceau-stained membrane used later for the detection of pERK 1/2 and GAPDH. The efficacy of the transfer of proteins onto a nitrocellulose membrane was checked using Ponceau staining. Images were captured by the camera of an Apple iPhone 7 plus. (B) Unmodified Western blot images of pERK 1/2 and GAPDH**.** Images were captured with the Odyssey CLx machine and exported with Image Studio 5.2.5 software. Bands in yellow boxes are shown in Fig. 6 as representative blots. Sham: sham-operated group, CKD: chronic kidney disease group, CKD+D1: chronic kidney disease group treated with the lower dose (13 μg/day, dose 1) of KISS1R agonist KP-13, CKD+D2: chronic kidney disease group treated with the higher dose (26 μg/day, dose 2) of KISS1R agonist KP-13, NC: negative control, L: left, R: right.


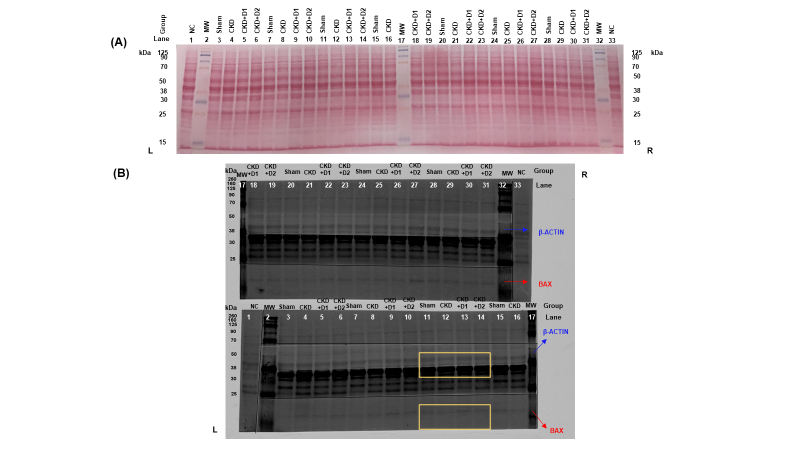


**Figure S8** Unmodified Western blot images of BAX and β-ACTIN. (A) Photo of the uncropped and unmodified Ponceau-stained membrane used later for the detection of BAX and β-ACTIN. The efficacy of the transfer of proteins onto a nitrocellulose membrane was checked using Ponceau staining. Images were captured by the camera of an Apple iPhone 7 plus. (B) Unmodified Western blot images of BAX and β-ACTIN. Images were captured with the Odyssey CLx machine and exported with Image Studio 5.2.5 software. Bands in yellow boxes are shown in Fig. 6 as representative blots. Sham: sham-operated group, CKD: chronic kidney disease group, CKD+D1: chronic kidney disease group treated with the lower dose (13 μg/day, dose 1) of KISS1R agonist KP-13, CKD+D2: chronic kidney disease group treated with the higher dose (26 μg/day, dose 2) of KISS1R agonist KP-13. MW: molecular weight marker, NC: negative control, L: left, R: right.

**
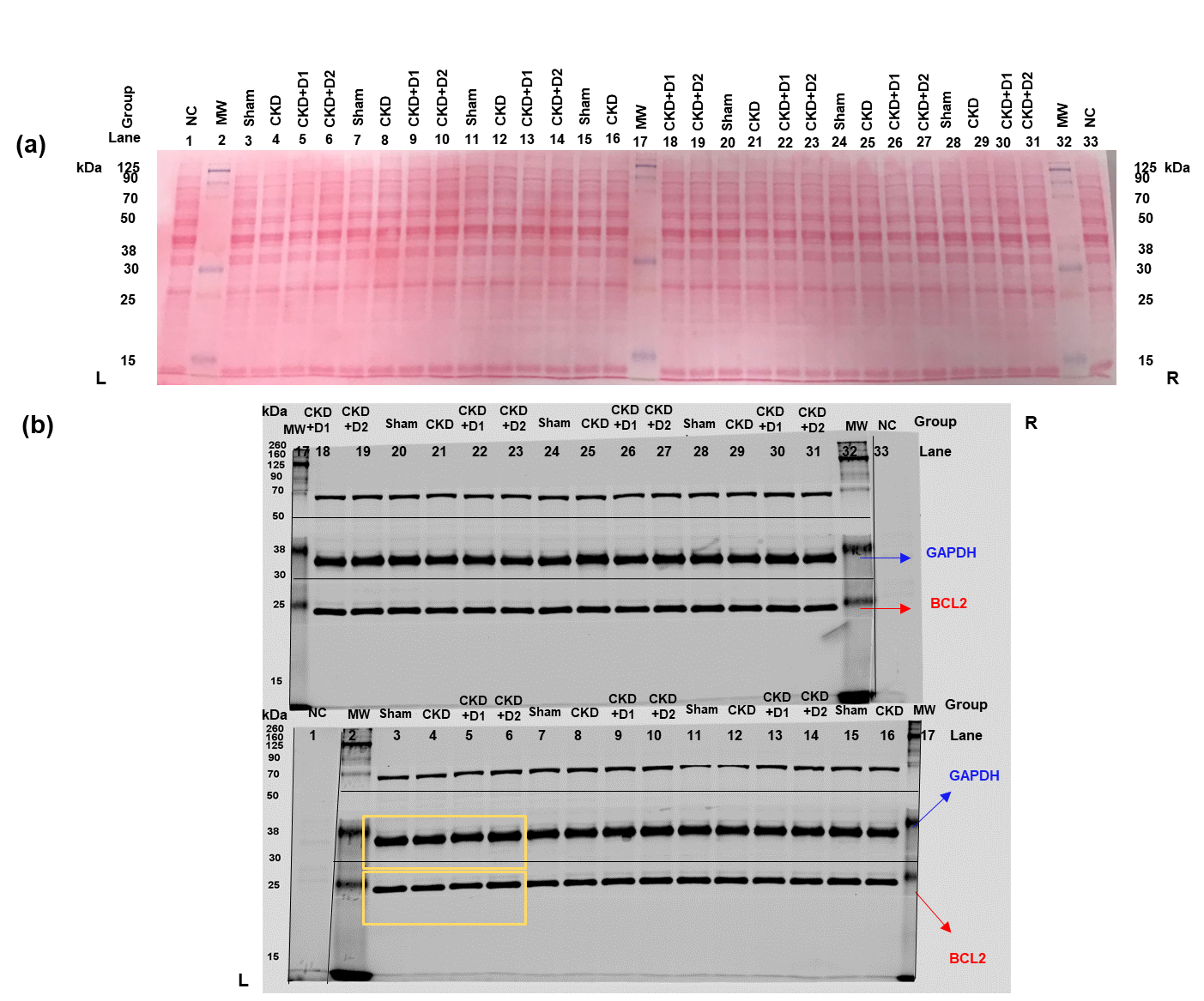
**

**Figure S9** Unmodified Western blot images of BCL2 and GAPDH. (A) Photo of the uncropped and unmodified Ponceau-stained membrane used later for the detection of BCL2 and GAPDH. The efficacy of the transfer of proteins onto a nitrocellulose membrane was checked using Ponceau staining. Images were captured by the camera of an Apple iPhone 7 plus. (B) Unmodified Western blot images of BCL2 and GAPDH. Images were captured with the Odyssey CLx machine and exported with Image Studio 5.2.5 software. Bands in yellow boxes are shown in Fig. 6 as representative blots. Sham: sham-operated group, CKD: chronic kidney disease group, CKD+D1: chronic kidney disease group treated with the lower dose (13 μg/day, dose 1) of KISS1R agonist KP-13, CKD+D2: chronic kidney disease group treated with the higher dose (26 μg/day, dose 2) of KISS1R agonist KP-13. MW: molecular weight marker, NC: negative control, L: left, R: right.

**
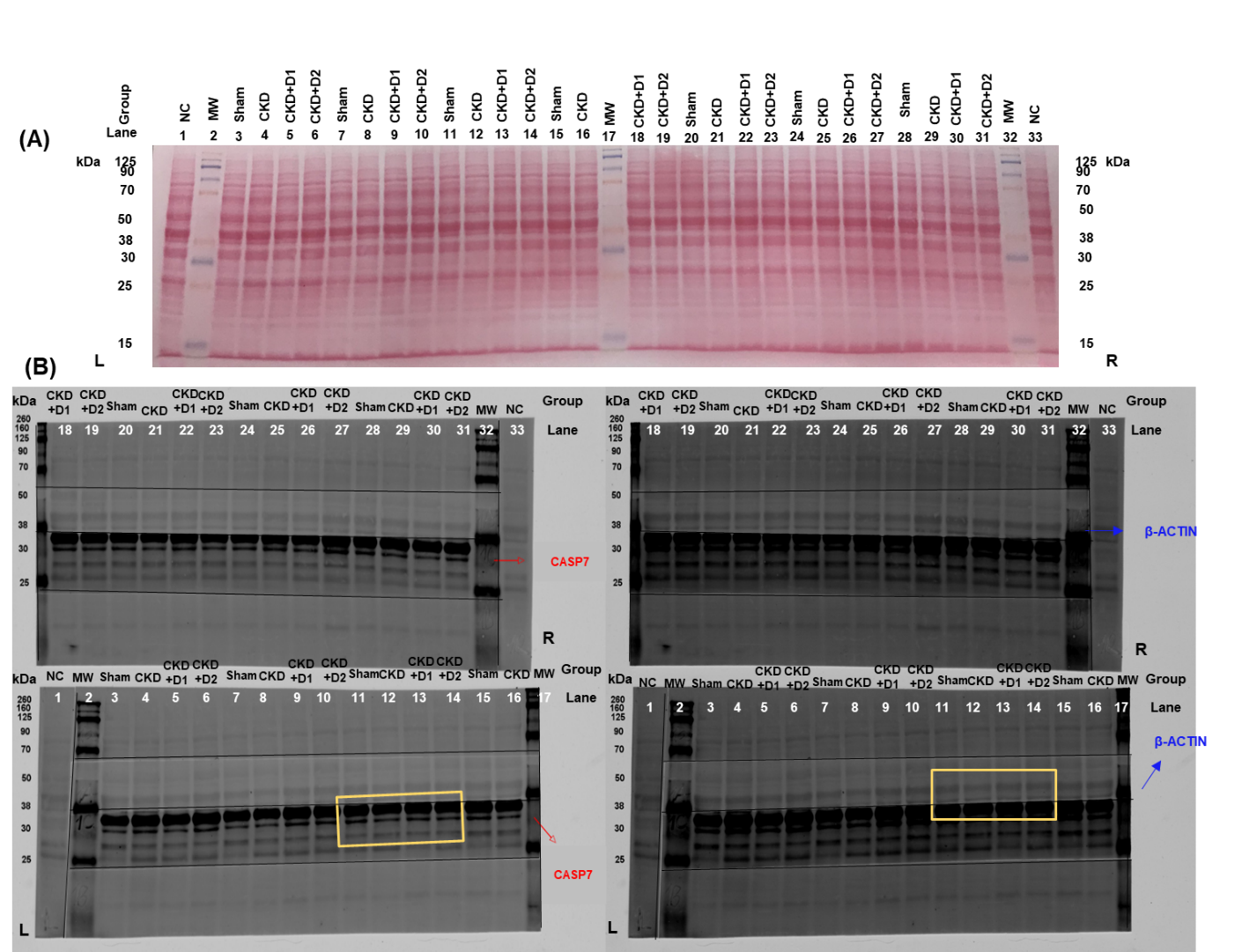
**

**Figure S10** Unmodified Western blot images of CASP7 and β-ACTIN. (A) Photo of the uncropped and unmodified Ponceau-stained membrane used later for the detection of CASP 7 and β-ACTIN. The efficacy of the transfer of proteins onto a nitrocellulose membrane was checked using Ponceau staining. Images were captured by the camera of an Apple iPhone 7 plus. (B) Unmodified Western blot images of CASP 7 and β-ACTIN. Images were captured with the Odyssey CLx machine and exported with Image Studio 5.2.5 software. Bands in yellow boxes are shown in Fig. 6 as representative blots. Sham: sham-operated group, CKD: chronic kidney disease group, CKD+D1: chronic kidney disease group treated with the lower dose (13 μg/day, dose 1) of KISS1R agonist KP-13, CKD+D2: chronic kidney disease group treated with the higher dose (26 μg/day, dose 2) of KISS1R agonist KP-13. NC: negative control, L: left, R: right.
